# The residue 86 of the Getah virus E2 glycoprotein mediates both glycosaminoglycan- and LDLR-dependent infection

**DOI:** 10.1371/journal.ppat.1014453

**Published:** 2026-07-31

**Authors:** Xiangshu Qiu, Rongguang Lu, Jiaxin Tian, He Zhang, Jiyong Zhou, Mengsi Sun, Xinyu Cao, Xiangyu Zhu, Bocheng Liu, Qihui Yu, Yuanyuan Li, Hualei Wang, Ningyi Jin, Huijun Lu, Ning Shi

**Affiliations:** 1 MOA Key Laboratory of Animal Virology, Zhejiang University Center for Veterinary Sciences, Hangzhou, China; 2 State Key Laboratory of Pathogen and Biosecurity, Changchun Veterinary Research Institute, Chinese Academy of Agricultural Sciences, Changchun, China; 3 Institute of Infectious Diseases, Shenzhen Bay Laboratory, Shenzhen, China; 4 State Key Laboratory for Diagnosis and Treatment of Severe Zoonotic Infectious Diseases, Key Laboratory for Zoonosis Research of the Ministry of Education, Institute of Zoonosis, and College of Veterinary Medicine, Jilin University, Changchun, China; Indiana University Bloomington, UNITED STATES OF AMERICA

## Abstract

Getah virus (GETV), a mosquito-borne alphavirus, poses an emerging threat to public health with its increasingly broad host spectrum. While glycosaminoglycans (GAGs) serve as critical attachment factors for many alphaviruses and the low-density lipoprotein receptor (LDLR) facilitates the cellular entry of several members, the precise viral determinants governing these interactions and their implications for viral virulence remain poorly defined. Here, we introduced an H86Y substitution, a potential adaptive mutation site, within the E2 glycoprotein of GETV using reverse genetics. The H86Y mutant replicated more efficiently in mosquito C6/36 cells but was consistently attenuated across several mammalian cell lines. In susceptible mouse models, H86Y infection led to reduced viral loads, milder histopathology, and lower inflammatory responses compared with the parental virus, yet still elicited robust protective immunity in adult mice. Mechanistically, a series of functional assays, including infection in GAG-deficient cells, decoy inhibition, co-immunoprecipitation, receptor overexpression and knockdown, and biolayer interferometry, demonstrated that the residue 86 in E2 glycoprotein is a critical determinant for GETV binding to both GAGs and LDLR. The H86Y mutation concurrently reduces these interactions, contributing the impairment of virus attachment and entry into mammalian cells. Furthermore, the GAG-binding site functionally overlaps with the LDLR interaction interface. In LDLR-deficient suckling mice, the impaired replication of H86Y persisted in examined tissues. However, pre-treatment with heparinase nearly completely eliminated this attenuation phenotype, further confirming that LDLR and GAG are the key host factors mediating attenuation phenotype for H86Y. In summary, the residue 86 of the GETV E2 glycoprotein represents a determinant of viral virulence, and an H86Y mutation attenuates GAGs and LDLR-dependent infection, providing mechanistic insights into alphavirus-host interactions and a potential target for antiviral and vaccine development.

## Author summary

Getah virus is an emerging mosquito-borne alphavirus. We investigated a naturally occurring substitution in the viral E2 glycoprotein, where histidine at position 86 is replaced by tyrosine. This single change, which we call H86Y, weakened the virus in mammalian cells and mice but unexpectedly enhanced its ability to infect mosquito cells. This host-specific switch may explain why the mutation persists in nature despite reducing disease severity in animals. Mechanistically, the attenuation was linked to impaired viral attachment and entry, resulting from a specific reduction in binding affinity for two critical host molecules, the attachment factor GAGs and the cellular entry receptor LDLR. Also, the GAG-binding site functionally overlaps with the LDLR interaction interface. Multiple mouse models supported that LDLR and GAGs are the key host factors mediating attenuation phenotype for H86Y. Despite being weakened, the mutant virus still triggered strong protective immunity, making it a promising vaccine candidate. Together, our findings identify E2 residue 86 as a key virulence determinant that modulates receptor usage, offering a precise target for antiviral and vaccine development against alphaviruses.

## Introduction

As globally expanding arboviruses, alphaviruses drive outbreaks of incapacitating human diseases and spillover epidemics in livestock with high socioeconomic burdens [1]. Getah virus (GETV), a single-stranded positive-sense RNA virus belonging to the *Alphavirus* genus of the *Togaviridae* family with Chikungunya virus (CHIKV), Sindbis virus (SINV), Eastern equine encephalitis virus (EEEV), which mainly transmitted by mosquitoes, is widely distributed across a broad geographical range [2,3]. Especially in China, since the initial report of GETV in 1956, the virus has spread to about 70% of provinces over the past seven decades [3–5]. In recent years, GETV has also been detected in wild boars, pangolins, and red pandas in China, indicating an increasingly broad host spectrum [6–8]. Furthermore, early serological surveillance revealed the presence of neutralizing antibodies against GETV in humans [9], indicating GETV presents a potential risk to humans.

The 70-nm virion surface of GETV is composed of 80 trimeric E2-E1 heterodimer spikes, which facilitate entry into host cells through endocytosis by binding to attachment factors and membrane receptors [10]. Heparan sulfate (HS), a negatively charged glycosaminoglycan (GAG), serves as an attachment factor for multiple alphaviruses [11–14]. Natural isolates of eastern equine encephalitis virus bind GAGs and use this interaction to promote neurovirulence [12]. However, efficient HS binding can reflect tissue culture adaptation rather than viral intrinsic property, such as CHIKV [11] and SINV [14]. While HS-mediated attachment enhances alphavirus infectivity *in vitro*, its effect on virulence *in vivo* varies, either increasing or decreasing pathogenicity depending on the virus and inoculation route [14,15]. The entry receptor, unlike attachment factors, not only concentrates viruses at the cell surface but also mediates virus internalization into cells. Recently, matrix remodeling associated protein 8 (MXRA8) has been identified as a functional receptor for several arthritogenic alphaviruses, including GETV, CHIKV, Mayaro virus (MAYV), Ross River virus (RRV), and O’ nyong nyong virus (ONNV) [16], whereas the low-density lipoprotein receptor (LDLR) serves as an entry receptor for multiple alphaviruses such as GETV, RRV, EEEV, Western equine encephalitis virus (WEEV), Semliki Forest virus (SFV), and Bebaru virus (BEBV) [17,18]. Multiple vector-borne viruses have evolved adaptive mutations over time to balance viral virulence and survival. Mechanically, such mutations might markedly alter viral binding to cell-surface attachment factors and/or receptors, modulating infectivity and pathogenicity as observed with E2-T179N in MAYV [19] and E2 residues 71–77 (K71A, K74A, K77A), 84–119 (R84A, R119A), and residues 156–157 (K156A, R157A), in EEEV [20].

**Fig 1:**
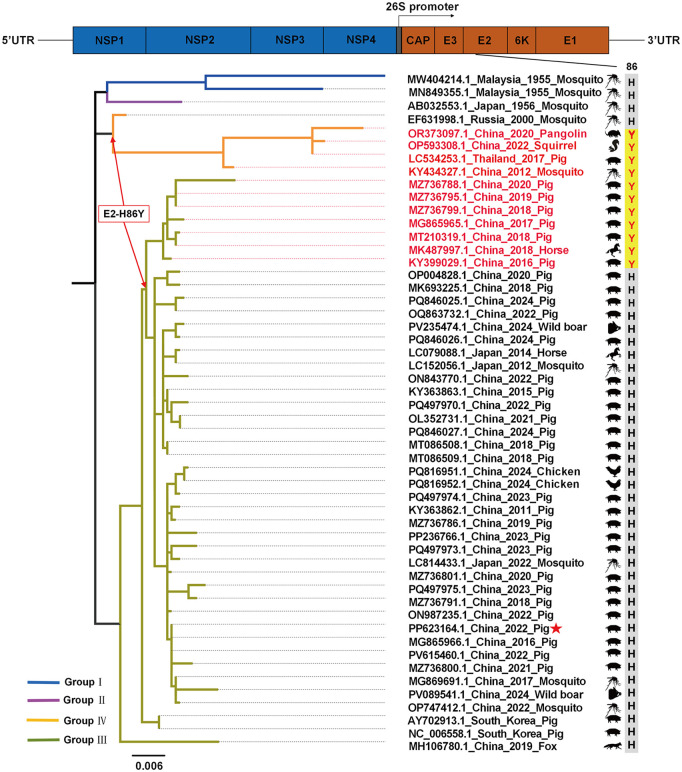
Phylogenetic analysis based on GETV E2 gene. The E2 nucleotide sequences of 48 representative GETV strains isolated from most of the reported countries were aligned and analyzed using the maximum likelihood method with 1,000 bootstrap replicates using the MEGA 12. The mutation (E2-H86Y) is colored in red and indicated at branch points of the phylogenetic tree. The parental strain contains a H86 in E2 protein used in this study, SD202206 (GenBank accession No. PP623164) is marked with red stars. Each GETV strain is named according to GenBank number, country, collection year, and host. The figure was created with FigureTree and Microsoft PowerPoint.

Partly consistent with results of previous studies [2,3,21], we performed adaptive evolution analysis of GETV E2 gene sequences and identified three potential adaptive mutation sites in E2 protein at residues 86, 323, and 368 (S1 and S2 Tables), among which E2-H86Y stood out as a convergent mutation appearing independently in two evolutionary lineages, Group Ⅳ and Group III (Fig 1). Epidemiological data show that strains carrying this substitution have been detected with increasing frequency since 2012 across multiple countries and host species, especially first reported GETV isolates or detection from dogs, horses, squirrels, and pangolins (S3 Table). The H86Y mutation replaces a positively charged histidine with a neutral tyrosine, reducing positive charge in a region known to influence GAG binding in other alphaviruses [11,20,22,23]. Despite these observations, the functional consequences of E2-H86Y for viral infection and pathogenesis remain unclear.

Here, we sought to test the hypothesis that the E2-H86Y of GETV serves as a key determinant of viral virulence. Using reverse genetics, we demonstrated that the H86Y mutation significantly attenuates GETV infection and replication in mammalian cells and viral virulence in mice by impairing binding to both GAGs and LDLR, while enhancing replication in mosquito cells. These findings identify E2 residue 86 as a key virulence determinant and provide new insights into the molecular basis of alphavirus-host adaptation.

## Results

### The effect of H86Y substitution on infection in cell cultures

**Fig 2:**
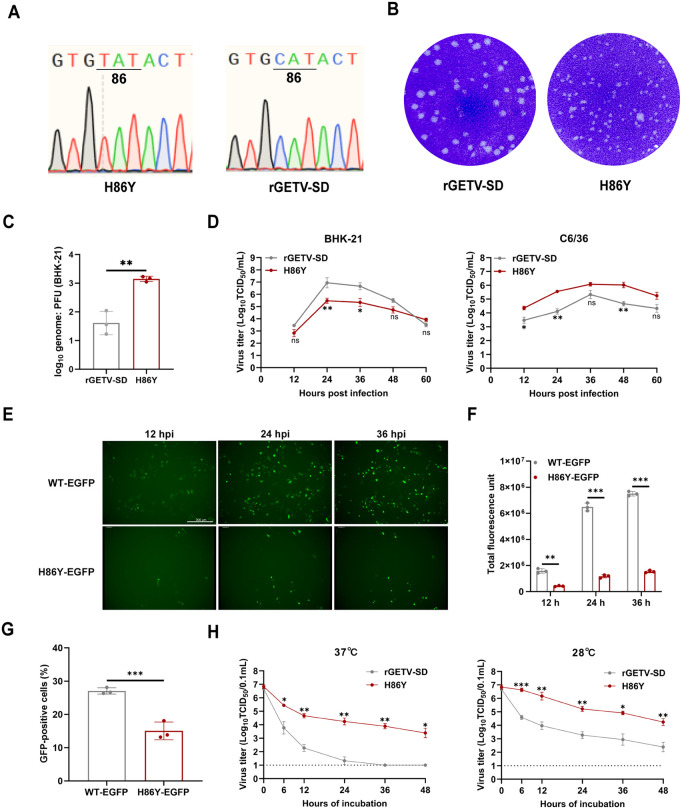
Characterization of the H86Y mutant. **(A)** Sequencing traces of E2 gene of rGETV-SD and H86Y viruses. **(B)** Plaque morphologies of rGETV-SD and H86Y viruses. **(C)** Genome-to-BHK PFU ratios for rGETV-SD and H86Y viruses. **(D)** Comparison of growth kinetics of rGETV-SD and H86Y viruses in BHK-21 (0.001 MOI) and C6/36 (0.01 MOI). **(E-F)** Live infected cell imaging and fluorescence calculation of ST cell inoculated with WT-EGFP or H86Y-EGFP at 0.01 MOI. Green color represents the EGFP expression in infected cells and calculated fluorescence signal using Image J. Scale bars, 300 μm. **(G)** The infectivity of WT-EGFP and H86Y-EGFP in ST cells. ST cells were inoculated with indicated virus at 0.01 MOI and processed for reporter gene expression by flow cytometry at 24 hpi. **(H)** Thermostability of viruses in cell-free environment at 37°C or 28°C. Data are presented as mean values ± SD at least three biological replicates (n = 3 independent experiments). Statistics were performed using unpaired Student’s t test (C, G); Two-way ANOVA (D, F, H). ns: not significant; * *P* < 0.05; ** *P* < 0.01; *** *P* < 0.001.

To investigate the effect of the E2-H86Y mutation in viral replication in cell culture, we introduced this mutation into the E2 protein of the prevalent GETV strain SD2206 (Figs 2 and S1A). After transfecting the infectious clone into Vero-E6 cells and sequencing verification, we successfully rescued two recombinant GETVs (rGETV): rGETV-SD and H86Y (Fig 2A and 2B). The engineered H86Y substitution remained stable without any reversion after 15 rounds of continuous passaging on BHK-21 cells (S4 and S5 Tables). Notably, H86Y exhibited smaller plaques in BHK-21 cells compared to rGETV-SD (Fig 2B). Cell‑adapted viral mutants with increased HS binding exhibit enhanced cell infectivity and a decreased genome‑to‑plaque‑forming unit (PFU) ratio [11,20]. Since the H86Y mutation replaces a positively charged histidine with a neutral tyrosine, we hypothesized that this loss of positive charge would weaken the mutant's binding to cell-surface GAGs such as HS, thereby reducing its replication in mammalian cells. To test this, we measured the genome-to-PFU ratios for both viruses on BHK-21 cells. The ratio for H86Y (~1400) was approximately 35-fold higher than the rGETV-SD (~40) (Fig 2C), suggesting a reduction in infectivity per particle. Considering the broad host range of GETV, we performed viral replication kinetics experiments in multiple cell lines derived from various hosts including hamster (BHK-21), african green monkey (Vero), swine (ST) and mosquito (C6/36). The results revealed that H86Y had a reduced proliferative capacity in three types of mammalian cells (Figs 2D and S2A). In contrast, the H86Y replicated more efficiently in C6/36 cells than the rGETV-SD. To enable real-time tracking of infection, we inserted an EGFP reporter gene downstream of the subgenomic promoter (N-terminal fusion to Cap protein), generating recombinant WT-EGFP and H86Y-EGFP viruses with comparable replication kinetics to their non-labeled counterparts (S1B and S2B Figs). Upon infecting ST cells, live-cell imaging result demonstrated that EGFP signal appeared later with less intensity in H86Y infected cells than rGETV-SD infected cells (Fig 2E and 2F; S1 Movie), supported by cytometry results (Figs 2G and S3). To confirm the genetic stability of H86Y, we performed whole-genome sequencing and sequencing depth analysis (S4 Fig) showed complete genome coverage with sufficient read depth, supporting reliable variant calling. Of note, single nucleotide polymorphism (SNP) and Sanger sequencing of viral supernatants from H86Y and rGETV-SD after 15 consecutive passages in BHK-21 revealed that H86Y virus did not acquire any revertant mutations at E2-86 residue (S6 and S7 Tables). The H86Y maintained its original cell phenotype throughout the passaging series.

Our previous report indicated the potential benefits of H86Y in enhancing the stability of viral proteins [2]. In this study, we incubated both viruses in cell-free suspensions at temperatures suitable for mosquitoes (28°C) and mammals (37°C). The viral titer of H86Y declined at a slower rate, extending the duration of viral activity at both temperatures (Fig 2H), implying that this mutation influences the virion's thermal stability. Above, in addition to improving viral thermal stability, the H86Y substitution in the E2 protein attenuated GETV replication in mammalian cells while enhancing it in C6/36 mosquito cells.

## The H86Y substitution attenuates virulence of GETV and diminished inflammation in susceptible mice models

**Fig 3:**
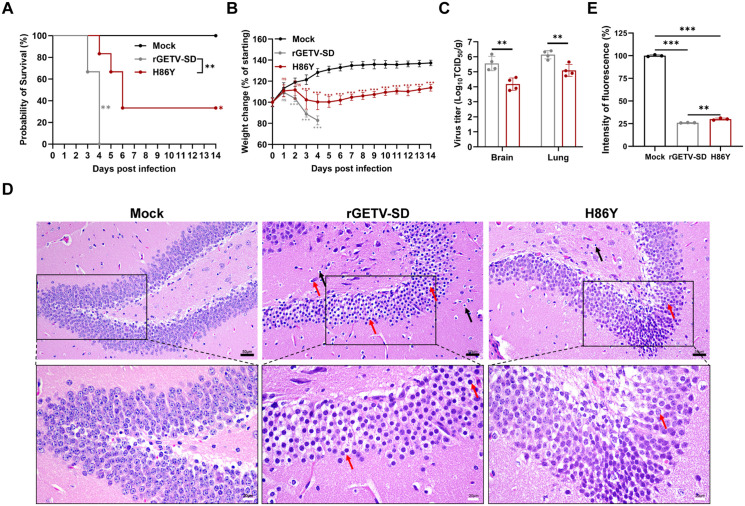
Effects of mutation at residue 86 for GETV virulence in A129 mice. Groups of six 6-week-old A129 mice were infected with 1 TCID_50_ rGETV-SD or H86Y virus or DMEM via footpad injection. Mice were monitored until day 14 (n = 6). **(A)** Survival curves. **(B)** Weight changes. **(C)** Virus titers of brain and lung at 2 dpi determined by TCID_50_. **(D)** Brain pathological change observed by HE staining. The red arrows represent the degenerated neuronal cells and the black arrows represent the glial cells. Black scale bars, 50 μm; white scale bars, 20 μm. Representative images were presented after similar results were obtained from at least 3 independent experiments. **(E)** Quantification of neuronal signals in A129 mouse brain. Relative neuronal fluorescence intensity was quantified using Image J software, normalized to the mock group. Data are presented as mean values ± SD at least three biological replicates (n = 3 independent experiments). Statistical significance was determined by Log-rank test (A), Two-way ANOVA (B), unpaired Student’s t test (C) or One-way ANOVA (E); ns: not significant; * *P* < 0.05; ** *P* < 0.01; *** *P* < 0.001.

To assess the impact of the H86Y mutation on virulence in vivo, A129 mice, which lack the type I interferon receptor gene (IFNAR1-KO) were used [24,25]. The LD_50_ of H86Y was significantly higher than that of rGETV-SD, indicating reduced virulence (Table 1). Furthermore, in mice individually challenged with a dose of 1 TCID_50_, the H86Y group showed a longer average survival times (ASTs) (6 days versus 4 days), a lower mortality rate (66.67% versus 100%), and a smaller decrease in body weight (Fig 3A and 3B). The H86Y variant showed markedly lower levels of infectious virus in mouse brains and lungs than those of rGETV-SD, with titers decreasing by approximately 10- to 20-fold at 2 days post-inoculation (dpi) (Fig 3C). HE staining revealed severe structural abnormalities in the dentate gyrus (DG) region of rGETV-SD-infected mice, characterized by widespread shrunken neuronal degeneration with pyknotic, hyperchromatic nuclei, enlarged perineuronal spaces, and a marked increase in surrounding glial cells. In contrast, H86Y infection resulted in comparatively milder pathological damage (Fig 3D). Similarly, immunofluorescence staining showed that rGETV-SD infection caused profound neuronal loss and obvious microglial activation, indicative of severe neuroinflammation and neuronal damage whereas H86Y mutant infection resulted in significantly milder neuronal depletion and microgliosis (Figs 3E and S5). No reversion accrued in infected H86Y A129 mice according to the SNP results (S6 and S7 Tables and S4 Fig). Collectively, these findings indicated that the E2-H86Y in GETV contributes to attenuated virulence in A129 mice.

**Table 1:** The comparison of LD_50_ of rGETV-SD and H86Y.

Virus	Dose (TCID_50_)	Survival (n)	Death (n)	Mortality (%)	ASTs (d)	LD_50_	Total LD_50_
**rGETV-SD**	100	0	6	100	2	0.10	0.10 ± 0.04
	10	0	6	100	3		
	1	0	6	100	4		
	0.1	3	3	50	6		
**H86Y**	100	0	6	100	2	0.44	0.39 ± 0.07^**^
	10	0	6	100	3		
	1	2	4	66.67	6		
	0.1	5	1	16.67	–		

** The LD_50_ of H86Y in A129 mice was significantly greater than that of the rGETV-SD in three independent experiments (n = 6) (unpaired t test, *P* = 0.0026).

**Fig 4:**
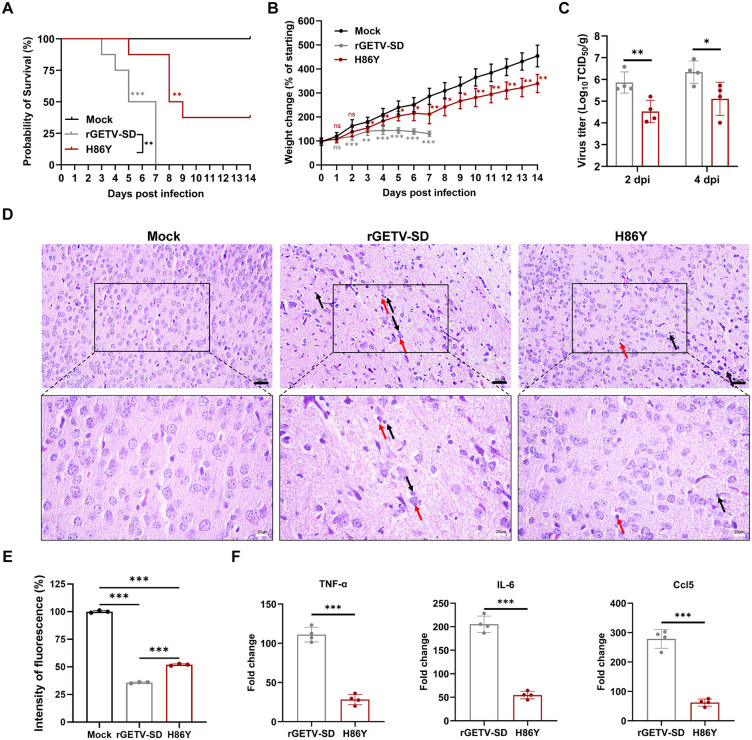
Effects of mutation at residue 86 for GETV virulence in 2-day-old C57BL/6J mice. 2-day-old C57BL/6J mice were infected with 25 μL of 10^4^ TCID_50_ rGETV-SD or H86Y by intracranial injection (i.c.) and groups injected with DMEM were used as control. Mice were monitored until day 14 (n = 8). **(A)** Survival curves. **(B)** Weight changes. **(C)** Virus titers of brains collected at 2 and 4 dpi. **(D)** Pathological changes by HE staining from the brain samples harvested at 4 dpi. Representative images were presented after similar results were obtained from at least 3 independent experiments. The red arrows represent the degenerated neuronal cells and the black arrows represent the glial cells. Black scale bars, 50 μm; white scale bars, 20 μm. **(E)** Quantification of neuronal signals in suckling mice brain (i.c.). Relative neuronal fluorescence intensity was quantified using Image J software, normalized to the mock group. **(F)** Fold change refers to gene expression of infected mice brains relative to mock-infected mice brains using RT-qPCR. Data are presented as mean values ± SD at least four biological replicates (n = 3 independent experiments). Statistical significance was determined by Log-rank test (A), Two-way ANOVA (B, C), One-way ANOVA (E) or unpaired Student’s t test (F). ns: not significant; * *P* < 0.05; ** *P* < 0.01; *** *P* < 0.001.

Neonatal suckling mice are susceptible to GETV and regarded as a reliable mouse model for assessing viral virulence [13,26]. To further assess the differences in virulence between rGETV-SD and H86Y, 2-day-old C57BL/6J mice were infected via intracranial (i.c.) or subcutaneous (s.c.) injection respectively. The suckling mice infected by H86Y exhibited obvious attenuated pathogenicity regardless of i.c. or s.c. (Figs 4 and S6–S8). All mice infected with rGETV-SD exhibited more serious neurological symptoms, minimal weight gain and succumbed to the infection within 7–10 dpi with ASTs of 5–6 days (Fig 4A and 4B). Conversely, only 50% and 60% mortality rates and the 8–9 days of AST were observed in suckling mice infected by H86Y via i.c. and s.c. route respectively (Figs 4A and S6A). Also, the viral titer of H86Y in brain tissue was significantly lower than that of rGETV-SD by nearly 10-fold at 2 dpi and 25-fold at 4 dpi (Figs 4C and S6C).

H&E and immunofluorescence staining further revealed that consistent with findings in A129 mice model, the pathological damage in the brain of H86Y infection were notably reduced compared to rGETV-SD infection (Figs 4D and 4E and S6-S8). These results clearly demonstrate that the E2-H86Y mutation attenuates virulence in a susceptible neonatal mice model.

To explore the underlying immune responses, we performed RNA sequencing on brain tissues from infected neonatal mice. In the context of weakened replication and reduced viral particles, the majority of responses induced by H86Y, which remained below the levels triggered by the rGETV-SD, involved genes closely associated with inflammatory and defense responses (S9 Fig). For example, a 3.1 to 4.5-fold reduction in multiple proinflammatory factor mRNAs (Ccl5, TNF-α and IL-6) was observed in mouse brains infected by H86Y (Fig 4F). These results suggest that the H86Y mutation reduces viral replication and induces weaker inflammatory responses in the brain.

### The mutation of E2-H86Y in GETV does not affect the induction of protective immunity in a mouse model

To investigate the impact of the H86Y mutation on the adaptive immune response, BALB/c adult mice were infected with either rGETV-SD or the H86Y (S10A Fig). As anticipated, mice infected with H86Y exhibited reduced viremia compared to those infected with rGETV-SD (S10B Fig). Despite this lower viral burden, H86Y-infected mice still mounted a robust seroconversion at 14 and 28 dpi (S10C and S10D Fig). IgG isotyping demonstrated that antibodies elicited by both viruses were predominantly of the IgG2a isotype (S10E Fig). Furthermore, to evaluate the immune response of mouse spleen lymphocytes after viral infection, we successfully expressed two virus-like particles (VLP), WT-VLP and H86Y-VLP, using 293F expression system (S11A–S11D Fig). Then upon stimulation with recombinant GETV-VLP proteins, spleen lymphocytes from mice immunized with either viruse showed enhanced proliferation and produced similarly high levels of IFN-γ and IL-4 (S10F–S10H Fig). Higher levels of IgG2a and IFN-γ indicates a Th1-biased immune response. Notably, after challenge with 10^5^ TCID_50_ of prevalent strain GETV SD2206, immunized mice did not develop detectable viremia and infectious virus in the spleen or ankle (S10I Fig).

Finally, to confirm whether the humoral immunity induced by H86Y immunization contributes to protection, pooled sera collected from immunized mice 28 days post-immunization were injected intraperitoneally into naive recipients. Recipients of immune serum showed significantly lower viral titers than controls (S10J Fig), indicating that neutralizing antibodies are essential to conferring protection. In conclusion, these results demonstrate that a single immunization elicits strong immune responses capable of protecting against high-dose GETV infection, and that the E2-H86Y mutation does not impair the induction of protective immunity.

### The H86Y mutation impairs virus binding and entry in mammalian cells

**Fig 5:**
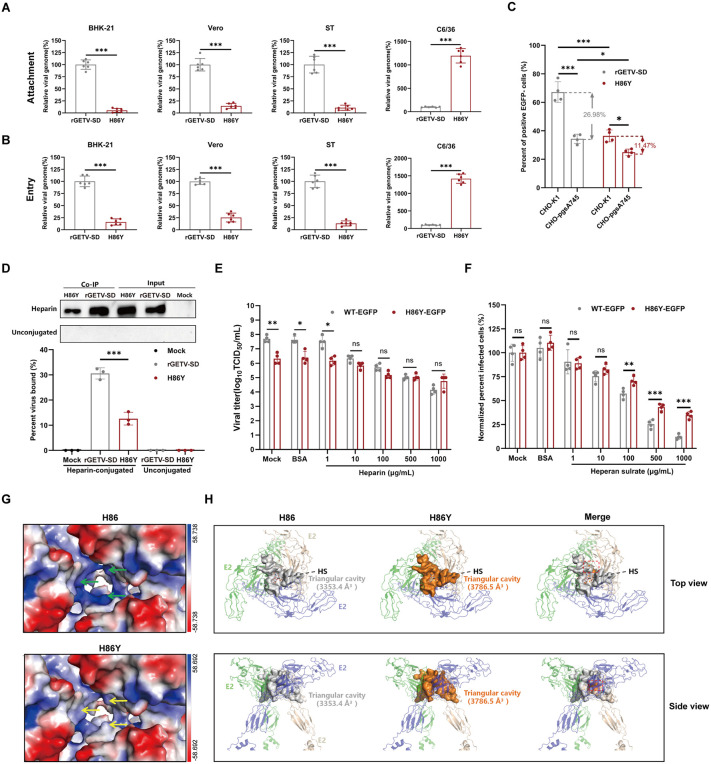
The E2-H86Y mutation confers less dependence on HS. **(A, B)** BHK-21, Vero, ST and C6/36 cells were incubated with equal genome copies of rGETV-SD or H86Y and then compared the difference of attachment (A) and entry (B). **(C)** The infection difference of CHO-K1 and CHO-pgsA745 quantified by flow cytometry. **(D)** The heparin-binding result of rGETV-SD or H86Y. **(E, F)** Neutralization of WT-EGFP or H86Y-EGFP by heparin determined using TCID_50_ (E) and flow cytometry (F). **(G)** Electrostatic potentials of rGETV-SD and H86Y E1/E2 trimers based on the cryo-EM structure of GETV (PDB ID: 7FD2). Positive potential appears in blue, while negative potential is shown in red. Green arrows mark the position of H86 in GETV-WT (top), and yellow arrows indicate the position of Y86 in H86Y (down). **(H)** Cavity detection and volume analysis. The Cryo-EM structure of heparin - EEEV (PDB ID: 6ODF) was used as the wild-type model and H86Y was generated by substituting residue His86 to Tyr. Means and SDs from at least three biological replicates are shown (n = 3 independent experiments). Statistical significance was determined by unpaired Student’s t test (A, B and D) and Two-way ANOVA (C, E and F). ns: not significant; * *P* < 0.05; ** *P* < 0.01; *** *P* < 0.001.

Given the reduced infectivity of H86Y in mammalian cells, we examined whether this defect occurred at the level of virus attachment or entry. When we incubated cells with equal genome copies of each virus, H86Y showed about 90% less viral RNA associated with cells after attachment and after internalization (Fig 5A and 5B), indicating that both steps were compromised. In contrast, in C6/36 mosquito cells, H86Y bound and entered more efficiently than the parental virus, consistent with its enhanced replication (Fig 2D).

We hypothesized that the reduced binding of H86Y to mammalian cells involves altered GETV‑GAG interactions. First, wild‑type CHO‑K1 and GAG‑deficient CHO‑pgsA745 cells were infected with WT‑EGFP or H86Y‑EGFP. Both viruses infected CHO‑pgsA745 cells less efficiently than CHO‑K1 cells, but the reduction was more pronounced for WT‑EGFP (27.0%) than for H86Y‑EGFP (11.5%) (Fig 5C). Then, equivalent genome copies of rGETV‑SD and H86Y were incubated with heparin‑conjugated beads. The proportion of bound virions was significantly lower for H86Y (12.6%) than for rGETV‑SD (30.5%) (Fig 5D). Finally, soluble heparin caused a dose‑dependent reduction in infection of Vero cells. However, this decrease was more pronounced for WT-EGFP, with the highest heparin concentration decreasing infectivity by about 3500-fold in viral titer and 80% in EGFP-positive cells respectively. In contrast, incubation of H86Y-EGFP with the same concentration resulted in only 38-fold decrease in viral titer and 65% decrease in EGFP-positive cells (Fig 5E and 5F). Collectively, these results confirm that GETV directly binds GAGs, and the H86Y mutation attenuates this interaction.

Using the published cryo-EM derived structure of GETV [27], we generated a putative structural model of the E1/E2 trimer containing the H86Y substitution. The three E2-86 residues likely occupy the inner apical surface of the cavity and the H86Y mutation probably reduces the positive charge and occupies more space within the cavity (Fig 5G). Since the only available cryo-EM structure of an alphavirus in complex with heparin is from EEEV [23], and given the high structural homology among alphavirus E2/E1 glycoproteins, we introduced the histidine to tyrosine substitution at E2-86 residue in GETV (corresponding to E2-84 in EEEV). The H86Y mutation may cause an ~ 13% enlargement of the C‑terminal triangular cavity (from 3353.4 Å³ to 3786.5 Å³), primarily near the upper region implicated in HS binding (Fig 5H). Overlay of the wild‑type and mutant cavities suggests an outward displacement of the pocket wall around residue 86, consistent with steric effects from the larger tyrosine side chain that may loosen inter‑subunit packing. These modeling data suggest that the E2‑H86Y mutation impairs GAG utilization and thus virus attachment. Of note, all structural comparisons are model‑based assumption and should be interpreted with caution.

### H86Y weakens the binding of GETV to LDLR but not to MXRA8

**Fig 6:**
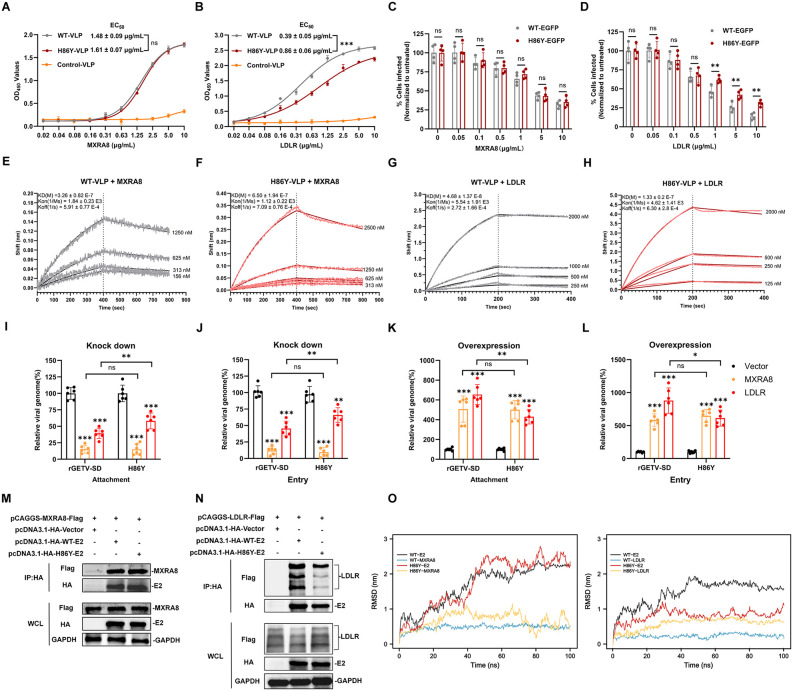
E2-H86Y mutation of GETV impairs the binding to LDLR receptors. **(A and B)** Binding of WT-VLP, H86Y VLPs or Control-VLP to mouse MXRA8 (A) or mouse LDLR (B) detected using ELISA. OD_450_, optical density at 450 nm. **(C and D)** Neutralization of WT-EGFP or H86Y-EGFP by MXRA8 (C) or LDLR (D) in Vero cells using flow cytometry. **(E-H)** Representative data of BLI with the binding and disassociation parameters of WT-VLP and H86Y-VLP with receptors. The MXRA8 binding KD between WT-VLP and H86Y-VLP has no statistically significant difference (*P* > 0.05). In contrast, the ~ 3-fold increase in LDLR binding KD between H86Y-VLP and WT-VLP is statistically significant (*P* = 0.037). **(I-L)** The differences in attachment and internalization of rGETV-SD or H86Y virus in BHK cells (10^5^ genomes/ cell), which knockdown or overexpress MXRA8/LDLR. **(M and N)** The E2-receptors binding capacity is detected by Co-IP assays in BHK-21cells using Western blotting. **(O)** Time-dependent RMSD of the E2 protein backbone and MXRA8 ligand or LDLR ligand in Wild-Type (WT) and H86Y mutant systems. Means and SDs from at least three biological replicates are shown (n = 3 independent experiments). Statistical significance was determined by unpaired Student’s t test (A, B, E, F, G and H) and Two-way ANOVA (C, D, E, F, I-L). ns: not significant; * *P* < 0.05; ** *P* < 0.01; *** *P* < 0.001.

Since the H86Y mutation weakened viral attachment and entry (Fig 6A and 6B), we wondered whether H86Y attenuates viral binding to cells by altering interaction with its entry receptors. We first conducted an Enzyme-linked immunosorbent assay (ELISA) using purified VLPs and concentration for 50% of maximal effect (EC_50_) was determined. Compared with the negative response of irrelevant receptor LDLRAD3 (S11E Fig) [17], both VLPs showed a dose-dependent enhancement response with mouse MXRA8 or LDLR. However, the EC_50_ values for WT-VLP (1.48 μg/mL) and H86Y-VLP (1.61 μg/mL) binding to MXRA8 showed no significant difference, whereas H86Y-VLP exhibited weaker interaction with LDLR, displaying an EC_50_ of 0.86 g/mL, more than double that of rGETV-SD with 0.39 μg/mL (Fig 6A and 6B). Subsequently, we performed an entry blocking assay using WT-EGFP and H86Y-EGFP in Vero cells. Increasing concentrations of MXRA8 or LDLR (0–10 μg/mL) dose-dependently reduced infection by both viruses, with no significant difference observed between the two strains in the presence of MXRA8 (Fig 6C). However, in LDLR competition assays, the H86Y mutation conferred a distinct advantage. While infection by both viruses was inhibited, the H86Y-EGFP exhibited a markedly attenuated decline compared to the WT-EGFP, particularly at the highest LDLR concentration, where approximately 30% of H86Y-EGFP-infected cells remained versus only about 15% for WT-EGFP (Fig 6D). Moreover, the results of biolayer interferometry receptor binding assay (BLI) showed no statistically significant difference between the measured equilibrium dissociation constant (*K*_D_) of MXRA8 for both VLPs (Fig 6E and 6F), but the *K*_D_ of LDLR for H86Y-VLP was 3.2-fold higher than that of the WT-VLP, indicating a significant decrease in binding affinity between H86Y and LDLR (Fig 6G and 6H).

Manipulating MXRA8 expression, either by over-expression or knockdown, did not significantly alter the difference between rGETV-SD and H86Y for attachment and internalization efficiency, indicating that differential interaction with MXRA8 does not explain the attenuated phenotype of H86Y (Figs 6I–6L and S12A). In contrast, LDLR knockdown reduced the attachment and internalization efficiency of H86Y (~40% and ~35%), a decrease substantially lower than that observed for rGETV-SD (~60% and ~55%) (Figs 6I, 6J and S12B). Conversely, LDLR over-expression significantly enhanced the viral attachment and internalization for both viruses, yet the increase for H86Y (~430% and ~600%) remained significantly lower than that for rGETV-SD (~650% and ~880%) (Fig 6K and 6L). These results suggesting the H86Y mutation on E2 protein result in a reduced dependence on LDLR for infection of cells by GETV.

Next, we investigated whether the E2 protein binds independently to the receptor protein and examined how the H86Y substitution influences this interaction. The results showed that both MXRA8 and LDLR could be pulled down by E2-HA infusion protein, indicating their ability to interact with the GETV-E2 protein (Fig 6M and 6N). Although no significant difference was observed in the MXRA8 band between WT-E2 group and H86Y-E2 group, the amount of LDLR pulled down by H86Y-E2 was significantly lower than the WT-E2, implying that the H86Y substitution reduces the interaction between GETV-E2 and LDLR.

Solvent-accessible surface area (SASA) and root-mean-square deviation (RMSD) were performed to assess ligand interface stability. For MXRA8, SASA distributions were comparable between WT and H86Y complexes (median ~35 nm²), indicating intact hydrophobic packing (S12C Fig). Ligand RMSD values in the WT complex remained consistently low (< 0.5 nm) and stable. In the H86Y complex, the MXRA8 ligand exhibited slightly increased flexibility with RMSD values primarily oscillating between 0.4 and 0.9 nm, yet it appeared to reach a new equilibrium toward the end of the simulation (Fig 6O, left panel). In contrast, the H86Y mutation markedly increased SASA for LDLR (from ~160 nm² to ~200 nm²) and expanded the interquartile range (IQR), reflecting the increased variability and fluctuating nature of the interaction compared to the WT (S12C Fig). RMSD analysis further revealed that while the LDLR ligand maintained a stable conformation in the WT complex (RMSD ~0.2–0.3 nm), it exhibited substantial positional instability in the H86Y mutant (~0.6–1.0 nm) despite a more stable protein backbone (Fig 6O right panel). Collectively, these data indicate that H86Y specifically destabilizes the GETV E2-LDLR interaction. Notably, SASA and RMSD analyses are model-based assumptions and should be interpreted with caution.

In summary, these results not only further confirms that both MXRA8 and LDLR are functional receptors for GETV but also suggest that the E2 glycoprotein can interact with these receptors. Importantly, the E2-H86Y mutation reduces the binding of GETV to LDLR, rather than MXRA8.

### The GAGs binding site in GETV overlaps with LDLR receptor interaction sites

**Fig 7:**
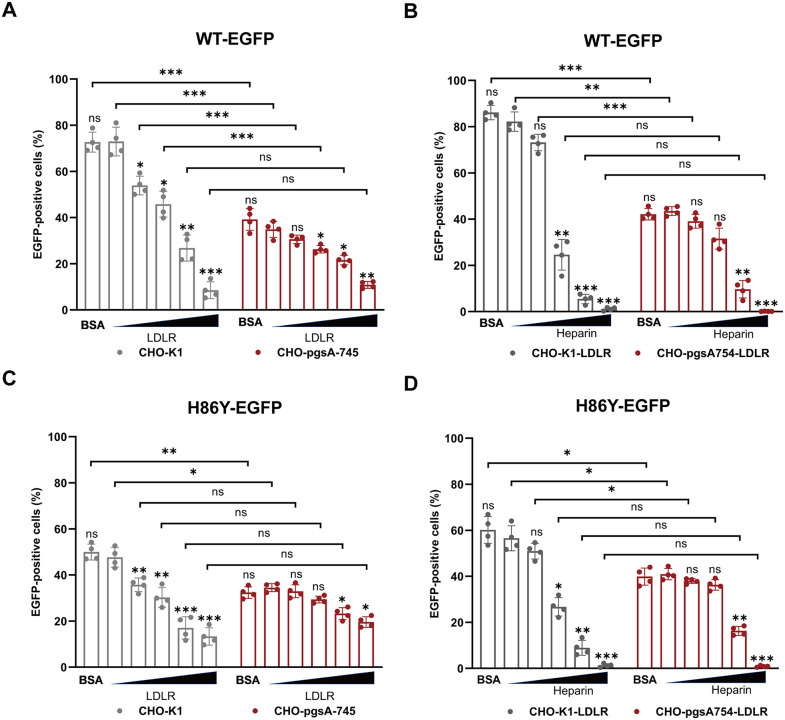
The GAGs binding site in GETV overlaps with LDLR receptor interaction sites. **(A and C)** Neutralization of WT-EGFP and H86Y-EGFP by LDLR (0, 0.5, 1, 10 µg/mL) in CHO-K1 and CHO-pgsA-745 cells by flow cytometry. **(B and D)** Neutralization of WT-EGFP and H86Y-EGFP by heparin (0, 10, 100, 500 and 1000 µg/mL) in CHO-K1 and CHO-pgsA745 which both over-expressing LDLR receptor by flow cytometry. Means and SDs from at least four biological replicates (n = 3 independent experiments). Statistical significance was determined by Two-way ANOVA. ns: not significant; * *P* < 0.05; ** *P* < 0.01; *** *P* < 0.001.

Given the dual weakening effect of the H86Y mutation on the interactions between GETV and GAGs, as well as between GETV and LDLR, we hypothesized that LDLR-mediated inhibition of GETV infection, may partly result from interference with GETV-GAGs binding. The CHO-K1 and CHO-pgsA745 cells hardly express LDLR (S12D Fig) or MXRA8 (S12E Fig) [16,20], thus differences in neutralization on these cells primarily be driven by cellular GAGs expression. The WT-EGFP was significantly neutralized by almost all LDLR decoy concentrations on both cell types. Especially at higher inhibitor concentrations, neutralization was markedly reduced on CHO-pgsA745 cells compared to CHO-K1 cells (Fig 7A). These findings indicate that LDLR-mediated neutralization is facilitated by blocking cellular GAG binding.

To assess whether heparin conversely diminishes GETV-LDLR interactions, we tested heparin neutralization of WT-EGFP on CHO-K1-LDLR cells that over-express LDLR (S12D Fig). The WT-EGFP was significantly inhibited by all concentrations of heparin, with stronger neutralization at higher concentrations (Fig 7B). To determine whether this effect was due to heparin blocking GETV-GAGs interactions to reduce overall attachment or directly interfering with LDLR engagement, we performed neutralization assays on CHO-pgsA745-LDLR cells, which lack GAGs but over-express LDLR (S12D Fig). The results showed heparin still inhibited WT-EGFP infection at high concentrations although to a lesser degree (Fig 7B). Similar trends were observed for the H86Y-EGFP in these assays (Fig 7C and 7D) though the inhibitory effect was attenuated to varying degrees.

MXRA8 neutralization occurred only at the highest concentration in CHO‑K1 cells and was absent in CHO‑pgsA745 cells, indicating HS‑independent neutralization (S13A Fig). In MXRA8‑overexpressing cells (S12E Fig), soluble heparin inhibited infection only in HS‑expressing CHO‑K1-MXRA8 cells, not in HS‑deficient CHO‑pgsA745-MXRA8 cells (S13B Fig). Thus, the inhibitory effect of heparin is mediated through HS‑dependent pathways rather than MXRA8.

Collectively, these results demonstrate that heparin contributes to GAG-mediated neutralization of GETV on GAG-positive, LDLR-overexpressing cells and could also compete with LDLR receptor binding.

### Impaired GAG binding is sufficient to attenuate H86Y

**Fig 8:**
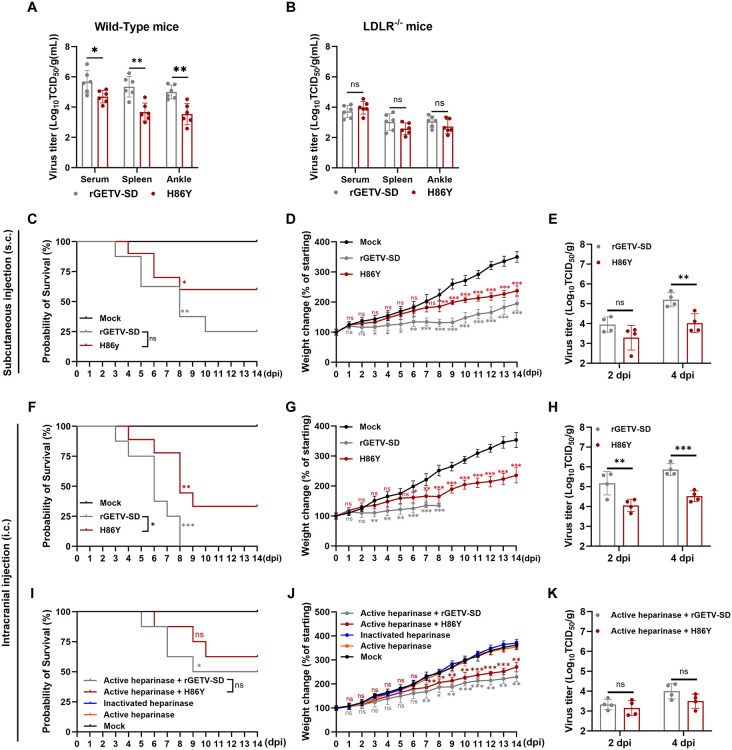
The infection results of rGETV-SD or H86Y in LDLR-deficient mice. **(A and B)** The viral titters in tissues of wild type adult mice (A) or LDLR^-/-^ adult mice (B) infected by rGETV-SD or H86Y. **(C-E)** 2-day-old LDLR^-/-^ mice were infected with 25 μL of 10^4^ TCID_50_ rGETV-SD or H86Y via s.c. inoculation and groups injected with DMEM were used as control. **(F-K)** 2-day-old LDLR^-/-^ mice treated with active heparinase (0.01U), heat-inactived heparinase or PBS were infected with 25 μL of 10^4^ TCID_50_ rGETV-SD or H86Y via i.c. inoculation. Mice were monitored until day 14. (C, F and I) Survival curves. (D, G and J) Weight changes. (E, H and K) Virus titers of brains collected at 2 and 4 dpi. Data are presented as mean values ± SD at least four biological replicates (n = 2 independent experiments). Statistical significance was determined by unpaired Student’s t test (A and B), Log-rank test (C, F and I), Two-way ANOVA (D, G, J, E, H and K). ns: not significant; * *P* < 0.05; ** *P* < 0.01; *** *P* < 0.001.

To dissect the relative contributions of GAGs and LDLR to H86Y attenuation, we performed infection assays using different kinds of mouse models. Both LDLR^-/-^ adult mice and wild-type adult mice, which exhibit similar baseline MXRA8 expression (S12G Fig), were infected by H86Y or rGETV-SD. The viral titer of H86Y was significantly lower than that of rGETV-SD by approximately 10- to 50-fold in wild-type adult mice (Fig 8A), but this difference almost disappeared in LDLR^-/-^ adult mice, despite the persistent differences in GAG binding (Fig 8B). It likely reflects the complicated effects of a mature immune system and functional redundancy among alternative entry receptors, which compensate for LDLR loss and mask the GAG-binding defect of H86Y. To overcome this limitation, we then utilized a more susceptible model, 2-day-old mice deficient in LDLR. We found that the suckling mice lacking LDLR infected by H86Y exhibited significantly attenuated pathogenicity regardless of s.c. (Fig 8C–8E) and i.c. (Fig 8F–8H). Compared with wild-type suckling mice, in the subcutaneous challenge model, mortality rates were reduced in both groups of virus-infected LDLR^-/-^ suckling mice. Nevertheless, the mortality rate of the H86Y group (40%) remained lower than that of the rGETV-SD group (70%) and viral titers in brain tissue showed a significant difference at 4 dpi. Similarly, all mice infected with rGETV-SD via i.c. showed minimal weight gain and 100% mortality, whereas only 66.6% of suckling mice infected with H86Y succumbed. Furthermore, viral titers in brain tissue were over 10-fold lower for H86Y than for rGETV-SD at both 2 dpi and 4 dpi.

To directly test whether impaired GAG binding alone is sufficient to attenuate H86Y, we conducted an in vivo HS degradation and infection experiment. The results showed that the survival rates of all mice in the non-infection groups were 100%, and no obvious clinical symptoms were observed. However, compared with the untreated infection mice, the mortality of rGETV-SD-infected mice pretreated with heparinase dropped to 50%, while that of H86Y-infected mice pretreated with heparinase decreased to 35%, with no significant difference in overall survival and weight change (Fig 8I and 8J). Besides, viral titers of rGETV-SD in the brain reduced approximately 70-fold, while those of H86Y decreased only 10-fold, reaching a comparable level with no significant difference (Fig 8K). Above, these findings reveal that GAG-binding defect of H86Y is sufficient to reduce GETV virulence, with LDLR engagement contributing to, but not solely responsible for the attenuation phenotype.

## Discussion

GETV, as a re-emerging mosquito-borne pathogen, has become an increasingly significant public health concern. Consequently, many researchers have focused on its genetic evolution and pathogenicity. Known adaptive sites include E2‑K253R involved in enhancing HS binding [13] and E2‑N262Q with improving MXRA8 receptor binding [28]. In this study, we identified three potential adaptive mutation sites and we found the E2-H86Y mutation reduced virulence for GETV *in vitro* and *in vivo* in mammals by down-regulating the binding of GETV to GAGs and LDLR. The single-amino-acid mutation might substantially affect virulence by modulating host antiviral and inflammatory responses, such as NSP2-K648A and NSP2-R649A in GETV [26] and E2-G55R in CHIKV [29]. Here, multiple susceptible mouse models, including A129 and two-day-old wild-type mice, demonstrated E2-H86Ymutation attenuated GETV replication with milder pathology, weaker antiviral and inflammatory genes, suggesting that lower viral loads may limit pathogen-associated molecular patterns exposure to innate immune sensors. Importantly, infection with H86Y still induced an adaptive immune response comparable in strength to that of rGETV-S, which effectively protected mice against subsequent GETV challenge. Similar phenomenon has been reported for GETV and other arboviruses [30,31]. Remarkably, H86Y exhibited greater thermal stability than rGETV-SD, supporting the predicted results of molecular dynamics [2], consistent with the stability effect in CHIKV [32,33]. Advantageously, the E2-H86Y mutation might facilitate the development of effective attenuated live vaccines suitable for long-term storage. Future research could focus on the combined application of H86Y and other similar amino acid mutations that cause weakened virulence in the development of pig attenuated vaccines [30].

To initiate infection, many alphaviruses first bind to target cells via GAGs (e.g., HS) [11,13,14], but the role of GAGs in alphavirus biology is complex. For many alphaviruses such as CHIKV and SINV, efficient HS binding often reflects tissue culture adaptation rather than an intrinsic property of naturally circulating strains. Wild-type SINV does not bind to HS, but BHK-21 passage selects for E2 mutations that confer HS binding [14]. Likewise, the attenuated CHIKV vaccine strain 181/25 exhibits enhanced GAG utilization driven by E2 residue 82 [11]. In contrast, NA-EEEV, a unpassaged strain, with high neurovirulence utilize HS as an attachment factor [12], and the GETV SD2206 strain used here (only two passages) inherently binds GAGs, a trait shared by other three GETV isolates [13]. The structural analysis suggested E2-86 residue is positioned at the E1/E2 trimeric interface, and substitution of H by Y at may increase the predicted cavity volume, yet experimental binding assays showed significantly reduced GAGs affinity and decreased the virulence.

A main feature of enhancing GAG‑mediated attachment is that it improves in vitro infectivity but increase or decrease in vivo virulence depending on the virus, mutation, and inoculation route. For EEEV, mutations at E2 residues 71, 74, and 77 reduce HS binding and in vitro infectivity, whereas wild‑type strains with high HS affinity exhibit attenuated pathogenicity via s.c. but enhanced neurotoxicity via i.c. [12,34]. For CHIKV (E2‑G82R) and SINV (E2‑K70), enhanced HS binding increases cell infectivity, reduces s.c. virulence by rapid blood clearance [11] and in SINV also increases i.c. neurovirulence [14]. For GETV, the E2‑K253R mutation enhances HS binding and cell infectivity but attenuates both s.c. and i.c. virulence by accelerating viral clearance from the circulation [13]. Differently, the H86Y mutation in this study reduces binding to both LDLR and HS, directly impairing viral attachment, internalization, and replication, leading to attenuation by both inoculation routes. These findings suggest a finely tuned balance in which either excessive or insufficient GAGs affinity disrupts GETV infection. Notably, a subset of mutations including SINV E2‑H55K70 [14], EEEV E2‑156/157 [20], and GETV H86Y show a positive correlation between GAG binding and virulence by both routes. Among these, the EEEV E2‑156/157 and GETV H86Y mutations simultaneously impair GAG binding and compromise protein receptor interactions, suggesting that a dual defect lowers the infectivity of a given dose, causing route‑independent attenuation. This functional coupling at key E2 residues highlights a subtle internal connection between GAG attachment and receptor engagement, positioning these sites as critical virulence determinants and attractive targets for rational vaccine design.

Interesting, the Co-IP results show that the GETV E2 protein alone pull down both MXRA8 and LDLR and E2-H86Y mutation markedly weakens E2 to LDLR. Although this appears to contrast with CHIKV, where MXRA8 engages a quaternary E1-E2 epitope [16,35], receptor binding modes among alphaviruses are diverse [36]. For instance, VLDLR binds exclusively to the E1 DIII domain of SFV [37], whereas the LDLR ligand-binding domain interacts with the E2-E1 spike of GETV [17]. Our findings extend this spectrum by demonstrating that the E2 subunit alone retains receptor-binding capacity, suggesting that core determinants reside in E2, while the full E2-E1 spike likely optimizes binding affinity and specificity for efficient viral entry.

In addition, in C6/36 mosquito cells, H86Y exhibited enhanced attachment, internalization, and replication, an unexpected contrast to its attenuated phenotype in mammalian cells. This host-dependent switch may explain the persistence of H86Y in natural GETV populations despite its attenuating effect in mammals. Mechanistically, mosquito cells lack LDLR [38] and possess HS with distinct structure and sulfation patterns from mammalian cells [39,40]. Thus, the H86Y-mediated reduction in HS and LDLR binding that impairs infection in mammalian cells may be inconsequential or even beneficial in mosquito cells, possibly by enhancing interaction with an unidentified mosquito-specific receptor [37]. This phenomenon aligns with the concept of host-specific fitness trade-offs in alphaviruses [41]. Similar observations exist for other alphaviruses and flaviviruses, such as E2-R82G in CHIKV, E2-T179N in MAYV and prM-H86R in DENV [11,19,42]. For instance, the MAYV E2-T179N mutation enhances mosquito cell replication and transmission while simultaneously attenuating replication and pathogenicity in mammals, illustrating an evolutionary trade-off where adaptation to the arthropod vector comes at the cost of reduced fitness in the vertebrate host. Together, these results support that H86Y might be a host-adapted mutation with different consequences in mammals versus mosquitoes, meriting further investigation.

Adult mice possess a fully developed interferon response and adaptive immunity to defense viral infection and the presence of other receptors, such as MXRA8 and the potential receptors LRP8 and VLDLR [17], might obscure the GAG-binding defect of H86Y in LDLR^-/-^ adult mice. Correspondingly, knockdown of MXRA8 in BHK-21 cells produced a stronger virological phenotype than knockdown of LDLR, suggesting that MXRA8 may serve as a dominant entry pathway. This observation warrants further investigation into the relative contributions of different entry receptors. We therefore evaluated H86Y in LDLR^-/-^ suckling mice, whose immune system is immature. H86Y remained significantly attenuated by both i.c. and s.c. inoculation compared to rGETV-SD. Since heparinase can eliminate HS on the cell surface [20,43], we treated the LDLR^-/-^ suckling mice with heparinase. Remarkably, the virulence of both viruses continued to decline but became comparable. These results demonstrate that impaired GAGs binding alone is sufficient to attenuate GETV in a susceptible suckling mouse model, independent of LDLR expression. The relationship between GAGs binding strength and *in vivo* virulence observed here is supported by SINV E2-H55K70 [14], which exhibits enhanced HS binding and increased virulence by both routes in mice.

To date, accumulating evidence indicates that the amino acid substitutions in alphavirus can alter viral binding and replication by simultaneously modifying viral binding to both attachment factors and receptors, or even by affecting interactions between virus and multiple receptors at once. For instance, E2‑D71A in CHIKV, a structure-guided mutation, reduces MXRA8 binding [44]. E1-M88L and E1-N20Y in CHIKV alter E2 conformation, heparin binding, and interactions with MXRA8 [45]. E2-T179N in MAYV decreases the dependence of human MXRA8 and LRP8 [19]. The mutagenesis of each of the three HS binding sites in EEEV (71–77, 84–119 and 156–157 in E2) either diminishes or abrogates interactions with VLDLR, LRP8, and LDLR receptors [20]. Importantly, these HS-binding sites on the E2 glycoprotein in EEEV overlap with protein receptor interfaces. Our data are consistent with this special mechanism. For GETV, LDLR decoy neutralization was reduced in GAGs-deficient cells and conversely, heparin neutralization in LDLR-overexpressing cells was diminished without GAGs, suggesting the GAG-binding site functionally overlaps with the LDLR-interaction interface on the E2 glycoprotein. Consequently, blocking one site also interferes with the other, and this interdependence is abrogated when GAGs are absent. This functional overlap likely represents a conserved alphavirus mechanism, wherein HS and protein receptor interactions are interdependent, shaping viral infectivity and virulence. Recently, a new study further showed that loss of N-linked glycans on the GETV E2 protein (particularly at residues N-200 and N-262) also reduces viral adsorption/entry and decreases virion binding to MXRA8 and LDLR in vitro, while simultaneously increasing heparin sensitivity/affinity [46], indicating an altered utilization of GAG attachment pathways and adding further support to the emerging concept that critical E2 residues serve as multifunctional hubs that coordinate interactions with attachment factors and entry receptors.

In conclusion, we found that the H86Y mutation significantly attenuates GETV infection and replication in mammalian cells and viral virulence in mice. In addition, E2-H86Y mutation reduces the attachment and entry of GETV to cells by down-regulating the binding ability of GETV to both HS and LDLR rather than MXRA8. Our study advanced the understanding of GETV pathogenesis and identified a promising target for attenuated vaccine and antiviral drug development, and provides insight into the molecular mechanisms underlying the increased prevalence of GETV.

### Limitations of this study

Several limitations should be acknowledged. First, the A129 and suckling mouse models used here, while susceptible to GETV and showing clear virulence differences, may not fully recapitulate natural disease progression in primary hosts (pigs, horses) [47,48]. Second, due to the absence of insectary facilities, we were unable to assess the role of the H86Y mutation in mosquito infection and transmission, which is an important aspect of the viral life cycle. Third, potential effects of H86Y on other possible receptors such as VLDLR, LRP8 remain unexplored [17]. Future studies in livestock and mosquito systems are needed to fully evaluate the attenuation phenotype and vaccine potential.

## Materials and methods

### Ethics statement

Animal experiments were conducted following guidelines for experimental animals’ welfare and ethics. All animal studies follow the protocols of Administrative Committee for Laboratory Animals of Changchun Veterinary Research Institute, Chinese Academy of Agricultural Sciences (approval number: IACUC of AMMS-11-2023-049).

### Phylogenetic analysis of GETV strains

The phylogenetic tree was constructed by aligning and analyzing the E2 nucleotide sequences of 48 GETV strains using the maximum likelihood method in MEGA 12, with 1,000 bootstrap replicates.

### Cell and virus

African green monkey kidney (Vero), swine testis (ST) and *Aedes albopictus* (C6/36) were maintained in Dulbecco’s Modified Eagle’s Medium (DMEM, Gibco) or MEM supplemented with 10% fetal bovine serum (FBS, Biological Industries) at 37°C with 5% CO_2_ when Baby hamster kidney (BHK-21) cells were maintained in DMEM with 5% FBS. Chinese hamster ovary (CHO-K1 and CHO-pgsA745) cells were maintained in Ham's F-12 medium (Gibco) with 10% FBS. A maintenance medium with 2% FBS was used for infected cells. The GETV SD2206 strain (GenBank: PP623164) used in this study was isolated from clinical porcine serum and subjected to only two passages in cell culture prior to infectious clone construction.

### Adaptive selection

116 available GETV E2 gene sequences were collected from the NCBI GenBank database (https://www.ncbi.nlm.nih.gov)up to December 1, 2025. To detect selections on the E2 gene, an ML tree based on the available sequences was reconstructed using Datamonkey (http://www. datamonkey.org). The methods used to investigate positive amino acid sites included Single Likelihood Ancestor Counting (SLAC), Fixed Effects Likelihood (FEL), Mixed EffectsModel of Evolution (MEME) and Fast Unconstrained Bayesian App Roximation for inferring selection (FUBAR). Significance levels were set with a p value threshold of 0.1 for SLAC, FEL and MEME, and while that of FUBAR was 0.9, we thought that a locus detected by more than two algorithms is positive selection, and used the method described by previous researchers to test the statistical significance.

### Establishing a reverse genetic system for GETV

To construct the infectious clone of GETV, we synthesized oligonucleotide DNA fragments containing specific restriction sites (cagctagcatcgactctagaatcgacttaattaaatcgactcgcgaatcgacagatctatcgacatttaaatatcgacgggcccatcgacatcgatatcgacgtttaaacatcgacgatccac) and cloned them into the pACYC-177 vector, yielding plasmid rpACYC. Using pcDNA3.1(+) and the full-length cDNA of GETV strain SD2206 as templates, we amplified the CMV promoter sequence along with segments S1–S5 with TransStart FastPfu Fly DNA Polymerase (TransGen Biotech) (S1 Table and S1 Fig). The S4-1 and S4-2 fragments were connected using overlap-PCR, while the Hepatitis delta virus ribozyme (HDVr) and bovine growth hormone (BGH) terminator fragment were synthesized with *Cla* I and *Pme* I restriction sites. After digestion and purification, the fragments were ligated into rpACYC to construct the infectious clone plasmids pGETV-SD. The E2-H86Y mutation was introduced by amplifying the target sequence with specific primers (at position 8778 of the corresponding nucleotide, a cytosine is substituted by a thymine.), followed by cloning into pGETV-SD using *BsaB* I and *Apa* I to generate pGETV-H86Y. Next, using overlap-PCR with primers from S4 Table, we introduced *Asc* I and *Xho* I sites downstream of the subgenomic promoter before the EGFP fragment, N-terminal fusion to Cap protein and amplified from pEGFP-N1, was cloned into pGETV-SD and pGETV-H86Y, yielding pGETV-WT-EGFP and pGETV-H86Y-EGFP. we inserted an EGFP reporter gene downstream of the subgenomic promoter. All recombinant plasmids were transformed into Stbl3 competent cells, and correctly sequenced plasmids were purified using the Endotoxin-free plasmid minikit I (OMEGA). For virus rescue, recombinant plasmids and an empty control plasmid were transfected into Vero cells using Lipofectamine 3000 (Thermo). Cells were maintained at 37°C until 80% exhibited cytopathic effects (CPE). The medium was subjected to a freeze-thaw cycle, centrifuged at 12,000 rpm for 30 min, and the supernatant was aliquoted and stored at -80°C. Viral titers were measured by TCID_50_ assay.

### Construction of plasmid encoding receptor proteins

The cDNAs of *Mesocricetus auratus* LDLR and MXRA8 were synthesized using total RNA isolated from BHK-21 cells as a template. To express receptor proteins with C-terminal Flag tags, their coding sequences were PCR-amplified using specific primer pairs (S5 Table) and cloned into the pCAGGS vector (NovoPro, V008798), yielding pCAGGS-MXRA8-Flag and pCAGGS-LDLR-Flag. Similarly, the E2 sequence was amplified using specific primers and either pGETV-WT or pGETV-H86Y recombinant plasmids as templates, then cloned into the pcDNA3.1-3× HA vector to generate pcDNA3.1-HA-E2-WT and pcDNA3.1-HA-E2-H86Y, which were confirmed by sanger sequencing.

### Plaque assays

Viral plaque assays were performed on confluent BHK-21 cell monolayers in 12-well plates using established protocols [17].

### TCID_50_ assay

Viral titers were determined by endpoint dilution assay on BHK-21 cells. Briefly, BHK-21 cells were seeded into 96-well plates at a density of 1 × 10^4^ cells per well and cultured overnight to reach approximately 80–90% confluence. Virus samples were serially diluted 10-fold in maintenance medium. Each dilution was added to eight replicate wells (100 μL per well). After incubation at 37 °C with 5% CO_2_ for 2–3 days, CPE were observed under an inverted microscope. The 50% tissue culture infectious dose (TCID₅₀) was calculated using the Reed–Muench method.

### Genome to PFU determination

rGETV-SD and H86Y viruses were titrated in triplicate by standard plaque assay on BHK-21 cells. Quantification of viral genomes was performed as previously described [49].

### Virus replication kinetics

BHK-21 (0.001 MOI), Vero (0.01 MOI), ST (0.01 MOI) and C6/36 (0.01 MOI) cells were infected with rGETV-SD or H86Y viruses at 37°C for 2 h. Subsequently the inoculum was then removed, and the cells were washed three times with PBS before being cultured for 60 h. Virus titers in the supernatant were measured every 12 h using the TCID_50_ assay.

### Real-time fluorescence assay

When ST was infected by WT-EGFP or H86Y-EGFP at 0.01 MOI, live imaging and visualization were performed using EVOS FL Auto2 Microscope (Invitrogen) for 36 h and calculated fluorescence signal using Image J.

### Flow cytometry

At the selected time points, cells expressing green fluorescence were trypsinized, resuspended in PBS, and quantified by flow cytometry (BD C6 Plus).

### Thermostability assay

Virus stocks were diluted to 10^7^ TCID_50_/mL in complete media and 200 μL aliquots were made. Aliquots were incubated in culture incubators at 28°C or 37°C. Infectious particle titers were determined by TCID_50_ at 0, 6, 12, 24, 36, and 48 h.

### Mouse experiments

Six-week-old A129 (Ifnar1-KO, I001199) mice were obtained from Cyagen (China). Groups of six mice (half female and half male) were inoculated with either rGETV-SD or H86Y at doses ranging from 0.1 to 100 TCID_50_ per mouse via footpad injection to compare the LD_50_, while control groups received an equivalent volume of DMEM. Survival, body weight, and clinical signs were monitored daily until 14 dpi. Additional groups of four mice were infected with 1 TCID_50_ of rGETV-SD or H86Y and euthanized at 72 hpi for brain and lung tissue collection that were used for TCID_50_ measurement, hematoxylin-eosin staining and immunofluorescence analysis.

Two-day-old wild type C57BL/6J mice from Huafukang Bioscience were infected through subcutaneous or intracerebral injections of rGETV-SD or H86Y (25 μL of 10^4^ TCID_50_ in DMEM). Both infected or mock-infected mice were observed daily for 14 days to record survival, weight changes, and clinical manifestations. In a parallel experiment, mice were euthanized at 2 dpi and 4 dpi, and their brain and lung tissues were collected, weighed, homogenized, and subjected to TCID_50_ titration. The brain tissues from infected or uninfected wild type suckling mice at 4 dpi also underwent hematoxylin-eosin staining, immunofluorescence analysis and RNA-sequence.

Four-week-old female BALB/c mice were inoculated subcutaneously with 10⁴ TCID₅₀ of rGETV-SD, H86Y, or DMEM. Serum samples were collected from the retro-orbital sinus at 1, 2, 3, 7, 14, and 28 dpi to assess viremia, neutralizing antibodies, and specific IgG antibody levels [50]. At 28 dpi, the same mice were challenged intraperitoneally with 10^_5_^ TCID_50_ of GETV strain SD2206. Two days post-challenge, the mice were euthanized, and viral titers in serum, spleen, and ankle tissues were measured using the TCID_50_ assay. In a separate experiment, mice were immunized following the same protocol. At 14 dpi, these animals were euthanized, and their spleens were harvested to evaluate the immune response of splenic lymphocytes via CCK-8 and ELISPOT assays as previously described [30]. Commercial ELISA kits (DAKEWE, 2210002 (IFN-γ), 2210402 (IL-4) were used to measure T cell cytokine responses following the manufacturer’s instructions, with both negative and positive controls included.

For passive transfer of neutralizing antibodies experiments, blood was collected from mice immunized with H86Y at 28 days post‑immunization. Sera were pooled, heat‑inactivated at 56°C for 30 min. Naive recipient mice (Four‑week‑old female BALB/c mice) were injected intraperitoneally with 100 μL of pooled immune sera or control sera (mock‑immunized) once daily for two consecutive days. 24 h later, all mice were challenged subcutaneously with 10^5^ TCID_50_ of GETV strain SD2206. At 2 days post‑challenge, mice were euthanized, and serum, spleen and ankle tissues were collected. Viral titers were determined by TCID_50_ assay.

Six-week-old female wild type C57BL/6J purchased from Huafukang and six-week-old female LDLR-knockout C57BL/6J mice (LDLR^-/-^, S-KO-02873) purchased from Cyagen, were infected with 10^5^ TCID_50_ of rGETV-SD or H86Y or PBS via intraperitoneal injection. All mice were euthanized at 2 dpi. The serum, spleens and ankles were harvested to quantify infectious titers via TCID_50._

Two-day-old LDLR^-/-^ mice were infected through subcutaneous or intracerebral injections of rGETV-SD or H86Y (10^4^ TCID_50_ in DMEM). In addition, heparinase mixture (heparinase I, II and III, H766343, Aladdin) was intracerebrally injected to LDLR^-/-^ suckling mice (0.01U). Heat-inactivated heparinase (100°C for 30 min), active heparinase and PBS served as control groups. 24 hours later, mice were challenged intracranially with 10⁴ TCID₅₀ of rGETV‑SD or H86Y. All mice were observed daily for 14 days to record survival and weight changes. In a parallel experiment, mice were euthanized at 2 dpi and 4 dpi, and brain tissue was collected, weighed, homogenized, and subjected to TCID_50_ titration.

### RNA-Seq and data analysis

The brain tissues from infected or uninfected 2-day-old wild type mice via i.c. at 4 dpi underwent RNA-seq (n = 4). Total RNA was extracted with TRIzol (Invitrogen, USA). (mRNA was isolated by Poly-T beads, fragmented, reverse-transcribed into cDNA and then was constructed to libraries by Beijing Novogene and performed Illumina sequencing. DESeq2 R package (1.42.0) analyzed differential expression between conditions, with significance thresholds set at padj ≤ 0.05 and |log2 (fold change)| ≥ 1. In addition, the PrimeScript RT kit (TaKaRa, Japan) converted RNA to cDNA, which was then quantified by qPCR using SYBR premix ExTaq (TaKaRa, Japan) with 2^−ΔΔCt^ analysis. The sequences data reported in this study was archived in the BIG Submission with the accession number CRA027116.

### Single nucleotide polymorphism (SNP) and Sanger sequencing

SNP on viral RNA extracted from brain tissues of H86Y-infected A129 mice and from BHK-21 cell supernatants following 15 serial passages was performed. Library preparation and deep sequencing were carried out on an Illumina platform (TPBio, China). Sequencing reads were aligned to the reference GETV genome (GenBank: PP623164), and a mutation is considered reliable only if its frequency exceeds 5%. In parallel, Sanger sequencing was conducted to confirm the stability of rGETV-SD and H86Y viruses after serial passaging. PCR products spanning the E2 region were sequenced by Sangon Biotech, China. Both methods confirmed that no reversion to the wild-type sequence occurred.

### Virus attachment and entry

For virus-binding assays, monolayer of adherent cells grown in a 24 well cell culture plate to the density of 1 million cells per well was incubated with equal genome copies (10^5^ genomes/cell) of rGETV-SD or H86Y virus and the cell plates were incubated at 4 °C for 1 h. After incubation, the inoculum was removed, and cell monolayers were washed three times with pre-chilled PBS to thoroughly remove unbound virions. Cells were then lysed directly in Trizol reagent (Invitrogen) for total RNA extraction. For internalization assays, the initial virus binding step was performed as described above. After removing unbound virions, pre-warmed complete medium supplemented with 2% FBS and 15 mM NH_4_Cl was added to each well, and plates were transferred to a 37°C or 28°C for 1 h. Then, cell monolayers were treated with proteinase K at a final concentration of 500 ng/mL for 2 h at 4 °C followed by three washes with pre-chilled PBS. Finally, for both assays, total RNA was extracted following the manufacturer’s instructions, and reverse transcription was performed using a gDNA-removing reverse transcription kit to synthesize cDNA. RT-qPCR was conducted using SYBR Green qPCR Master Mix targeting the GETV E1 gene and normalized to housekeeping gene (S5 Table). In parallel experiments, BHK-21 cells were either transfected with pCAGGS-MXRA8-Flag or pCAGGS-LDLR-Flag using Lipo3000 (Thermo) for 24 h, or transfected with siMXRA8 or siLDLR using RNAiMAX (Thermo) for 24 h, before performing the virus attachment and internalization assays described above.

### Infection of CHO-K1 and CHO-pgsA745

CHO-K1 and CHO-pgsA745 cells were incubated with WT-EGFP or H86Y-EGFP at 1 MOI for 1 h. The inoculum was removed, and complete medium supplemented to contain 20 mM ammonium chloride was added to prevent subsequent rounds of infection. After incubation at 37 °C for 18 h, the infected cells were then trypsinized, resuspended in PBS, and analyzed by flow cytometry (BD C6 Plus) to quantify positive cells.

### Inhibition of GETV infection with soluble HS

WT-EGFP or H86Y-EGFP was incubated with soluble heparin (Sigma) or bovine serum albumin (BSA) (Sigma) at 4°C for 30 min. Vero (0.1 MOI), CHO-K1-LDLR (1 MOI) and CHO-pgsA745-LDLR (1 MOI) were then infected with the pretreated viral strains for 1 h at 37°C. Following removal of the inoculum, the cells were maintained in complete medium containing 20 mM ammonium chloride to block secondary infection. After 18 h of incubation at 37°C, infected cells were detached with trypsin, washed with PBS, and analyzed by flow cytometry (BD C6 Plus) to quantify EGFP-positive populations or the cell supernatant was used to determine the viral titters by TCID_50_.

### Heparin-conjugated magnetic beads binding assay

An equal amount of heparin- and unconjugated magnetic beads (BEAVER) were utilized according to the manufacturer's instructions. Elution samples were used for western blot with poly-clonal antiserum (1: 2000, prepared in our laboratory) against GETV-E2. The density of protein bands in the blot was determined and the percentage of virus bound to beads was calculated as the mean of bound virus divided by the total input virus.

### Production and purification of GETV virus-like particles (VLPs)

The structural polyprotein gene Cap-E3-E2-6K-E1 was amplified using pGETV-SD and pGETV-H86Y plasmids as templates and cloned into the pcDNA3.4(+) vector via *Xba* Ⅰ and *Cla* Ⅰ restriction sites. Following sequence verification, both plasmids were transfected into 293F cells using TA-293 transfection reagent (K20001, KAIRUI biotech) according to the manufacturer’s protocol. The methods for collecting and purifying VLPs are described in reference [17]. Freshly prepared VLPs were stored at 4°C for immediate use for ELISA and biolayer interferometry assays.

### Enzyme-linked immunosorbent assay (ELISA)

ELISA was perform using WT-VLP or H86Y-VLP (10 μg/mL) and mouse LDLR-His, MXRA8-His protein or LDLRAD3-hFc (TargetMOI) (0.02 - 10 μg/mL) with control-VLP (Kactus Biosystems) as negative control as described in reference [17].

### Biolayer interferometry receptor binding assay (BLI)

Mouse LDLR was biotin-labeled using EZ-Link NHS-LC-LC-biotin Kit (Thermo) and purified by Zeba Spin Desalting Columns (Thermo). The binding affinity between GETV and receptor was determined by GatorPrime (Gator) at 25°C. The receptor protein diluted 2-fold (2.5 μM) and the VLP at a concentration of 1 μM were loaded onto the biosensor to determine the association kinetics. The association and dissociation period time are 400 s.

### Entry blocking assays

WT-EGFP or H86Y-EGFP were incubated with serially diluted MXRA8-His or LDLR-His proteins (0, 0.05, 0.1, 0.5, 1, 5, or 10 μg/ml) for 1 hour at 37°C before adding the mixture to adherent Vero (0.1 MOI), CHO-K1-LDLR/MXRA8 (1 MOI) and CHO-pgsA745-LDLR/MXRA8 (1 MOI) in 96-well plate. After 1 h incubation at 37°C, we washed the cells three times with PBS and replaced the medium with DMEM containing 1% FBS and 20 mM ammonium chloride to block secondary infection. The percentage of EGFP-positive cells was quantified by flow cytometry at 12 hpi.

### Co-IP assays

BHK-21 cells were plated in 6-well plates and transfected with 2 μg of either pcDNA3.1-HA-WT-E2 or pcDNA3.1-HA-H86Y-E2 with pCAGGS-MXRA8-Flag or pCAGGS-LDLR-Flag plasmids using Lipo3000 (Thermo) following the manufacturer's protocol. At 24 hpi, the cells were rinsed three times with ice-cold PBS and lysed in 0.2 mL of IP lysis buffer (P1003, Beyotime) for 30 min on ice. Following centrifugation, the supernatant was incubated with anti-HA magnetic beads (88836, Thermo) according to the manufacturer’s protocol. The immunoprecipitated proteins were resolved by 10% SDS-PAGE and analyzed via Western blotting.

### Electrostatic energy, cavity detection and volume analysis

The structure of the GETV (PDB ID: 7FD2) was used to analyzed for electrostatic surface potentials by PyMOL. For cavity detection and volume analysis, the Cryo-EM structure of the heparin - EEEV (PDB ID: 6ODF) was used as the WT model. Cavity detection and volume analysis were performed using parKVFinder with default parameters and KVFinder with automatically identified multiple cavities within the trimeric structure [51].

### Molecular dynamics simulations

All MD simulations were performed using GROMACS 2020.6 with the AMBER99SB-ILDN force field. Protein–ligand complexes were solvated in cubic TIP3P water boxes (1.0 nm solute-box buffer) and neutralized with Na^+^/Cl^-^ counterions. Each system underwent energy minimization, equilibration, and a 100 ns production run. Long-range electrostatics were computed via the PME method, hydrogen-related bond lengths were constrained by the LINCS algorithm, and a 2 fs timestep was applied. RMSD and SASA analyses were carried out using native GROMACS tools.

### Statistical analysis

All data were analyzed using the Prism 9 software (GraphPad) and are presented as mean  ±  standard deviation. All experiments were performed in two to three independent replicates with at least three technical replicates per group.

## Supporting information

S1 Fig The diagram of recombinant plasmids construction. **(A)** The strategy for the construction of a full-length CMV-launched infectious clone based on the GETV isolate, SD2206. A *XhoI* site in the genome was silenced as the genetic marker to rescue the virus. **(B)** The strategy for the construction of an infectious clone of GETV with EGFP gene downstream of the subgenomic promoter (N-terminal fusion to Cap protein).
(TIF)

S2 Fig The replication kinetics of recombinant GETV in Vero, ST and BHK-21 cells. Data are presented as mean values  ±  SD at least three biological replicates (n = 3 independent experiments). Statistics were performed using Two- way Anova; ns: not significant; * *P* < 0.05; ** *P* < 0.01; *** *P* < 0.001.
(TIF)

S3 Fig The percent of positive infected ST cells determined by Flow cytometry. ST cells were inoculated with WT-EGFP or H86Y-EGFP at 0.01 MOI or DMEM. EGFP-positive cells were counted using flow cytometry at 24 hpi (n = 3 independent experiments).
(TIF)

S4 Fig Sequencing depth of H86Y mutant virus. Whole-genome sequencing depth of H86Y from brain tissues of H86Y-infected A129 mice and from BHK-21 cell supernatants following 15 serial passages. The high, uniform coverage supports reliable variant calling.
(TIF)

S5 Fig Immunofluorescence analysis of Neuron and microglia in the brains of A129 mice infected by rGETV-SD, H86Y, or PBS. Six-week-old A129 mice were inoculated via footpad injection with 1 TCID₅₀ of rGETV-SD, H86Y mutant virus, or PBS (Mock). Brain tissues were collected at 3 dpi and processed for immunofluorescence staining. Neurons were labeled with anti-Neun antibody (red), microglia with anti-Iba1 antibody (green), and cell nuclei with Dapi (blue). Scale bar: 50 μm. Representative images were presented after similar results were obtained from two independent experiments.
(TIF)

S6 Fig Effects of mutation at residue 86 for GETV virulence in 2-day-old C57BL/6J mice via subcutaneous injection (s.c.). 2-day-old C57BL/6J mice were infected with 25 μL of 10^4^ TCID_50_ rGETV-SD or H86Y by subcutaneous injection and groups injected with DMEM were used as control. Mice were monitored until day 14 (n = 8). **(A)** Survival curves. **(B)** Weight changes. **(C)** Virus titers of brains collected at 2 and 4 dpi. **(D)** Pathological changes by HE staining from the brain samples harvested at 4 dpi. Representative images were presented after similar results were obtained from two independent experiments. The red arrows represent the degenerated neuronal cells and the black arrows represent the glial cells. Black scale bars, 50 μm; White scale bars, 20 μm. **(E)** Quantification of neuronal signals in suckling mice brain (s.c.). Relative neuronal fluorescence intensity was quantified using Image J software, normalized to the mock group. Data are presented as mean values ± SD at least four biological replicates (n = 3 independent experiments). Statistical significance was determined by Log-rank test (A), Two-way ANOVA (B, C), One-way ANOVA (E) and unpaired Student’s t test (F). ns: not significant; * *P* < 0.05; ** *P* < 0.01; *** *P* < 0.001.
(TIF)

S7 Fig Immunofluorescence of Neuron and microglia in the brains of suckling mice infected by rGETV-SD, H86Y, or PBS via intracranial injection (i.c.). Neurons were labeled with anti-Neun antibody (red), microglia with anti-Iba1 antibody (green), and cell nuclei with DAPI (blue). Scale bar: 50 μm. Representative images were presented after similar results were obtained from t two independent experiments.
(TIF)

S8 Fig Immunofluorescence of Neuron and microglia in the brains of suckling mice infected by rGETV-SD, H86Y, or PBS via subcutaneous injection (s.c.). Neurons were labeled with anti-Neun antibody (red), microglia with anti-Iba1 antibody (green), and cell nuclei with DAPI (blue). Scale bar: 50 μm. Representative images were presented after similar results were obtained from two independent experiments.
(TIF)

S9 Fig RNA-seq analysis. **(A and B)** Volcano plots indicating differentially regulated genes of rGETV-SD or H86Y infected mouse brains. **(C and D)** Top 15 Gene Ontology terms of up-regulated genes in rGETV-SD or H86Y versus mock-infected brains. **(E)** Heatmap analyses of expression of genes related to the inflammatory response. Heatmap shows Z-score normalized expression; red indicates above mean, blue below mean.
(TIF)

S10 Fig Comparison of immunogenicity between rGETV-SD or H86Y in BALB/c mice. BALB/c adult mice were infected with 10^4^ TCID_50_ of rGETV-SD or H86Y virus or DMEM via subcutaneous injection. At 28 dpi, the mice were subcutaneously challenged in the same site with 10^5^ TCID_50_ of prevalent strain GETV SD2206 (n = 8). On day 2 post-challenge, the mice were euthanized. **(A)** Scheme of vaccination and challenge. **(B)** Mice viremia after infection. **(C)** Neutralizing antibody titers in sera at 14 dpi and 28 dpi determined using prevalent strain GETV SD2206. **(D)** GETV-specific IgG titers in serum at 14 and 28 dpi measured by ELISA performed using inactivated rGETV-SD or H86Y by formaldehyde and presented as OD_450_ values. **(E)** The OD_450_ values of IgG2a and IgG1 isotypes in sera of rGETV-SD or H86Y infected mice. **(F-I)** Two weeks after immunization, mouse spleens were collected, spleen cells were isolated and restimulated with recombinant GETV VLP protein (n = 4). **(F)** Specific splenocyte proliferation. RPMI 1640 medium was used as a negative control, and concanavalin A was used as a positive control. The stimulation index (SI) was calculated using the formula SI = (OD _stimulant_ - OD _1640_) / (OD _control_ - OD _1640_). **(G and H)** The spleen lymphocytes secreting IFN-γ and IL-4 were quantified by ELISPOT with both negative and positive controls. **(I)** Viremia and viral titers in spleen and ankle at 2 dpi. Means and SDs from at least four biological replicates are shown. **(J)** Passive transfer of immune serum protects mice from GETV challenge. Pooled sera collected from H86Y-immunized mice at 28 days post-immunization were injected intraperitoneally into naive recipient mice and control recipients received serum from mock-immunized mice once daily for two consecutive days. 24 hours later, all recipients were challenged subcutaneously with 10^5^ TCID_50_ of GETV-SD2206. Viremia and viral titers in spleen and ankle at 2 dpi were determined by TCID_50_ (n = 2 independent experiments). Statistical significance was determined by Two-way ANOVA (B, C, D, F, G and H), unpaired Student’s t test (E) and Mann-Whitney test (J); ns: not significant; * *P* < 0.05; ** *P* < 0.01; *** *P* < 0.001. Panel A in this figure was created with Adobe Illustrator.
(TIF)

S11 Fig Expression and identification of H86Y-VLP and WT-VLP. **(A and B)** Proteins present in purified VLPs of GETV were separated using western-blot with anti-GETV PcAb and anti-GETV-E2 PcAb polyclonal antibody. Molecular masses are indicated in kDa. PcAb, polyclonal antibody. **(C and D)** Electron micrographs of negatively stained purified VLPs of GETV. Scale bar is 50 nm. **(E)** ELISA-binding of WT-VLP, H86Y-VLP or control VLP to irrelevant receptor LDLRAD3. Representative images were presented after similar results were obtained from two independent experiments.
(TIF)

S12 Fig Western-blot analysis of MXRA8 and LDLR expression in various cell lines and in tissues from both wild-type and LDLR-deficient mice. **(A, B)** BHK-21 cells were either transfected with siMXRA8 or siLDLR for 24 h and then identified by Western-blot. **(C)** Boxplots representing the distribution of ligand SASA over the simulation trajectories. **(D-E)** The endogenous expression level and over-expression level of LDLR or MXRA8 in CHO-K1 and CHO-pgsA745 cells. **(F)** The endogenous expression level of LDLR in tissues from both wild-type mice and LDLR-deficient mice. The experiment was performed twice with similar results, and representative micrographs are shown.
(TIF)

S13 Fig The GAGs binding site in GETV does not overlap with LDLR receptor interaction sites. **(A)** Neutralization of WT-EGFP by MXRA8 (0, 0.5, 1, 10 µg/mL) in CHO-K1 and CHO-pgsA-745 cells by flow cytometry. **(B)** Neutralization of WT-EGFP by heparin (0, 10, 100, 500 and 1000 µg/mL) in CHO-K1 and CHO-pgsA-745 which both over-expressing MXRA8 receptor by flow cytometry. Means and SDs from at least four biological replicates (n = 3 independent experiments). Statistical significance was determined by Two-way ANOVA. ns: not significant; * *P* < 0.05; ** *P* < 0.01; *** *P* < 0.001.
(TIF)

S1 Table Adaptive selection sites of GETV-E2 protein. (DOCX)

S2 Table Adaptive mutation sites of the GETV-E2 protein in different studies. (DOCX)

S3 Table 31 sequences of GETV containing the E2-Y86 mutation were identified. (XLSX)

S4 Table Sequences of the primers for constructing infectious clone of GETV. (DOCX)

S5 Table Sequences of the primers used for PCR. (DOCX)

S6 Table The sequencing results of H86Y and rGETV-SD. (DOCX)

S7 Table Statistics of depth and coverage information of H86Y. (DOCX)

S1 Movie The real-time monitoring of WT-EGFP or H86Y-EGFP infection at 0.01 MOI in ST cells until 36 hpi. (MP4)

S1 Raw Images Source data file including original uncropped and unadjusted images underlying all blot and gel results. (PDF)
