# Supplementary figures and images for: The residue 86 of the Getah virus E2 glycoprotein mediates both glycosaminoglycan- and LDLR-dependent infection
Source: PLoS Pathog. 2026 Jul 31;22(7):e1014453. doi: 10.1371/journal.ppat.1014453 (PMC13426916; doi:10.1371/journal.ppat.1014453)

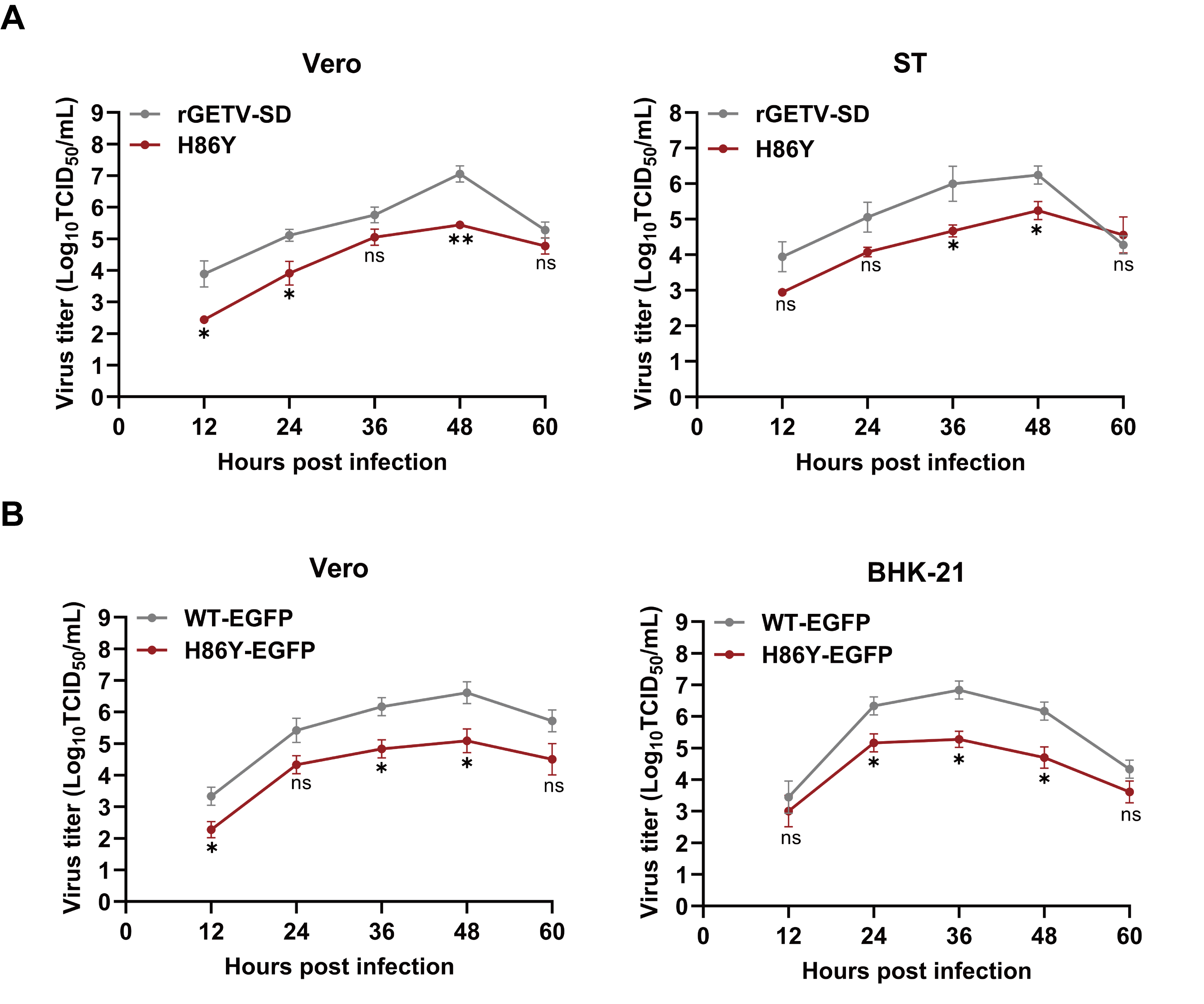

Supplement: S2 Fig — Data are presented as mean values ± SD at least three biological replicates (n = 3 independent experiments). Statistics were performed using Two- way Anova; ns: not significant; * P < 0.05; ** P < 0.01; *** P < 0.001. (TIF) [file ppat.1014453.s002.tif]

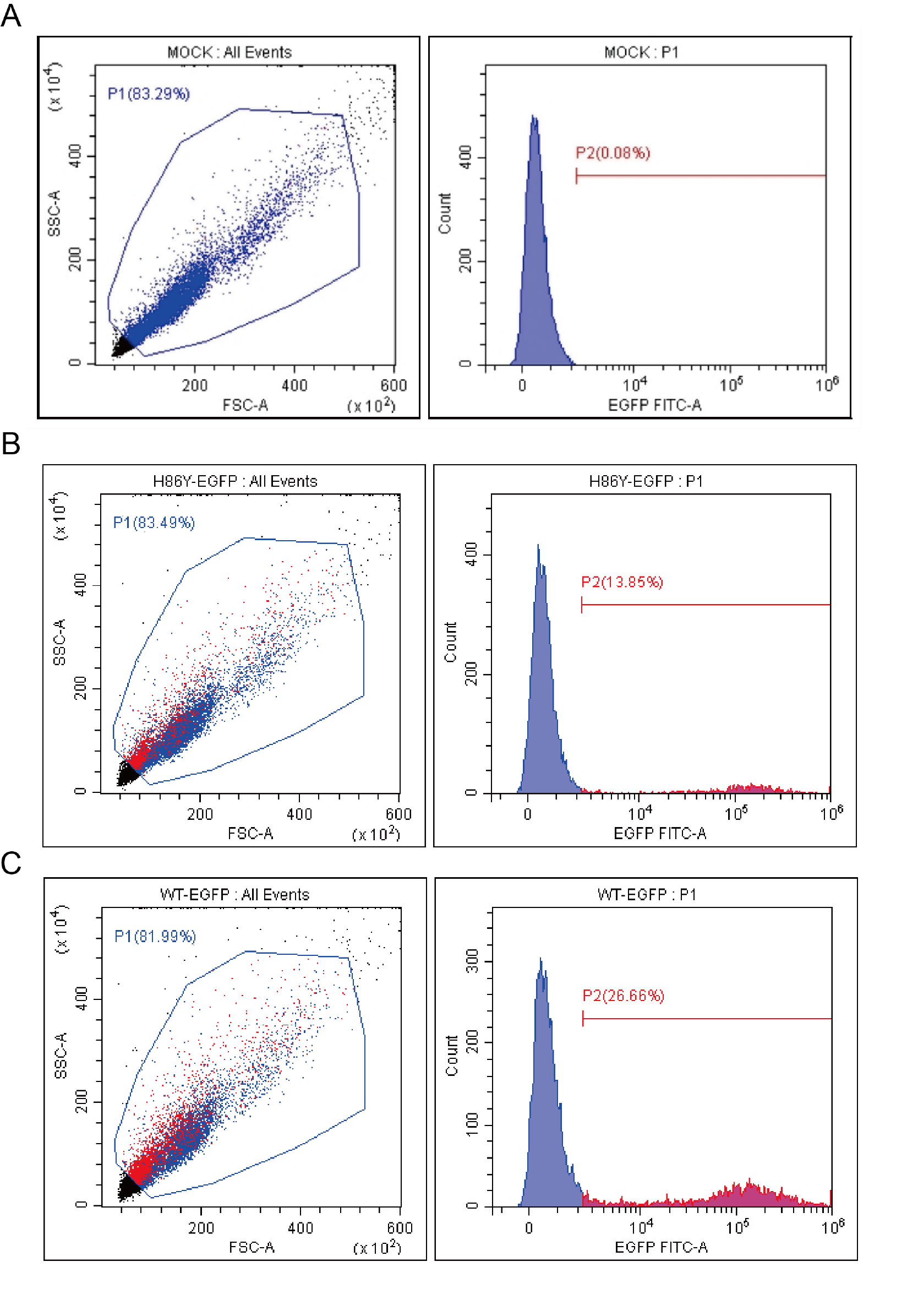

Supplement: S3 Fig — ST cells were inoculated with WT-EGFP or H86Y-EGFP at 0.01 MOI or DMEM. EGFP-positive cells were counted using flow cytometry at 24 hpi (n = 3 independent experiments). (TIF) [file ppat.1014453.s003.tif]

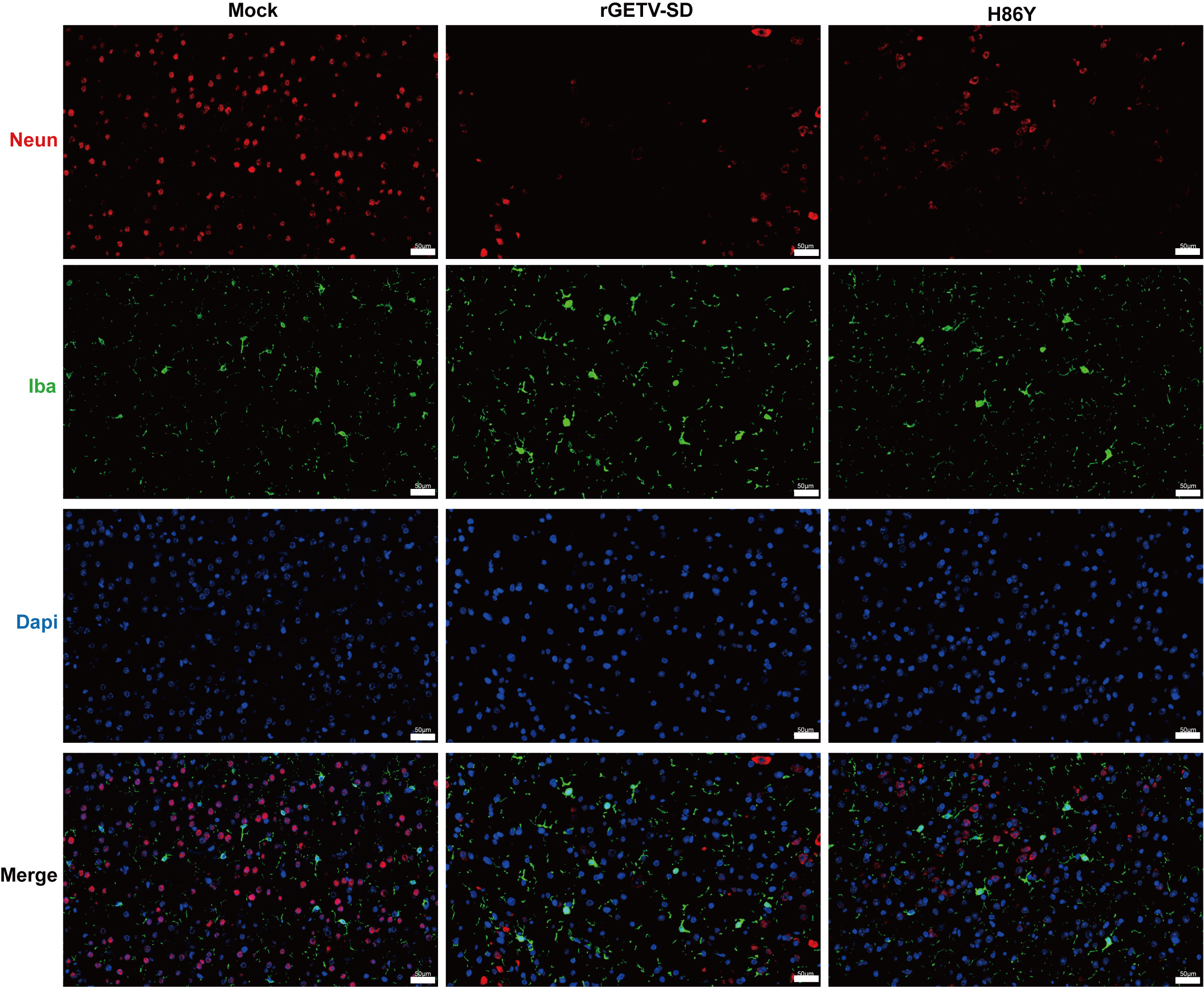

Supplement: S5 Fig — Six-week-old A129 mice were inoculated via footpad injection with 1 TCID₅₀ of rGETV-SD, H86Y mutant virus, or PBS (Mock). Brain tissues were collected at 3 dpi and processed for immunofluorescence staining. Neurons were labeled with anti-Neun antibody (red), microglia with anti-Iba1 antibody (green), and cell nuclei with Dapi (blue). Scale bar: 50 μm. Representative images were presented after similar results were obtained from two independent experiments. (TIF) [file ppat.1014453.s005.tif]

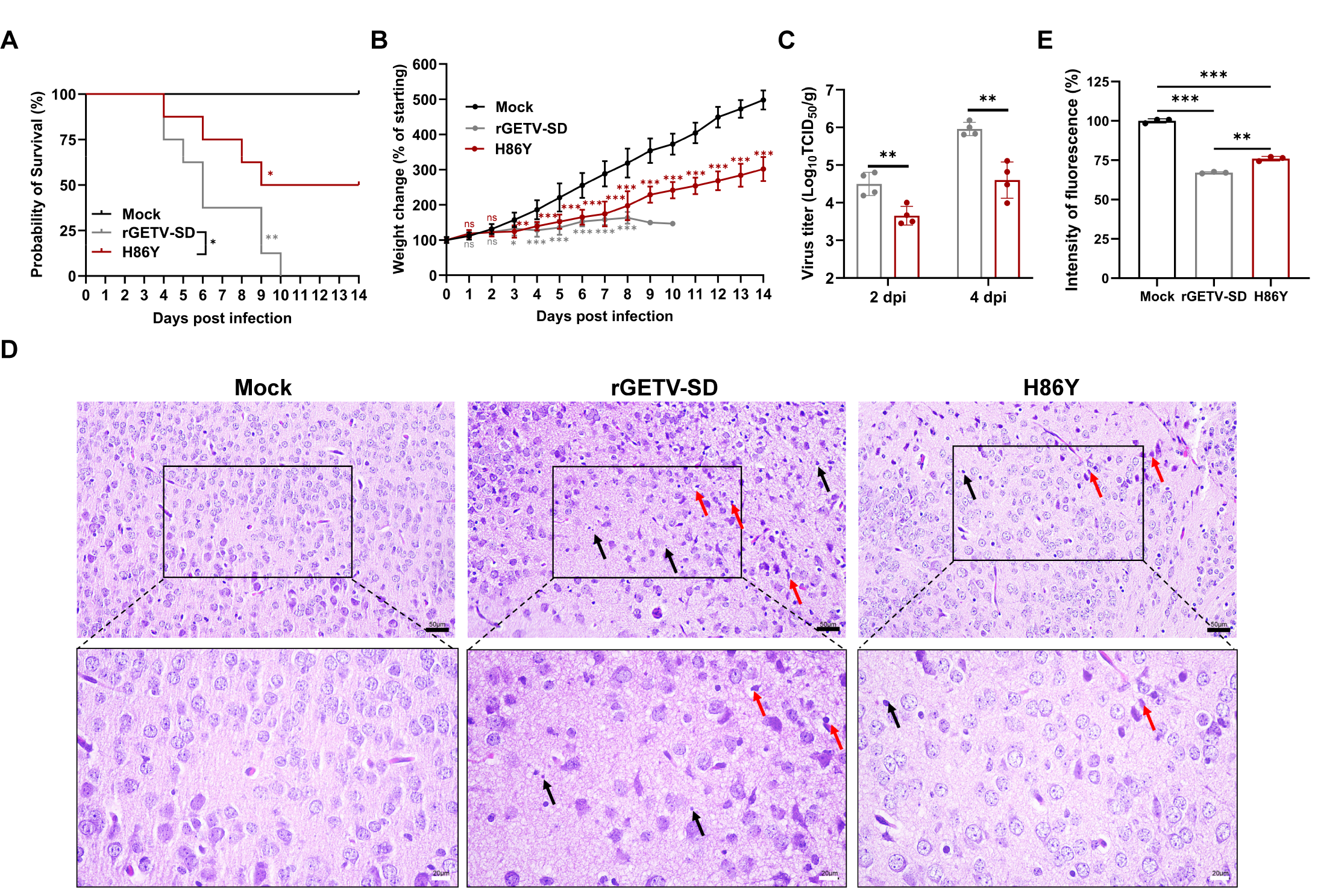

Supplement: S6 Fig — 2-day-old C57BL/6J mice were infected with 25 μL of 104 TCID50 rGETV-SD or H86Y by subcutaneous injection and groups injected with DMEM were used as control. Mice were monitored until day 14 (n = 8). (A) Survival curves. (B) Weight changes. (C) Virus titers of brains collected at 2 and 4 dpi. (D) Pathological changes by HE staining from the brain samples harvested at 4 dpi. Representative images were presented after similar results were obtained from two independent experiments. The red arrows represent the degenerated neuronal cells and the black arrows represent the glial cells. Black scale bars, 50 μm; White scale bars, 20 μm. (E) Quantification of neuronal signals in suckling mice brain (s.c.). Relative neuronal fluorescence intensity was quantified using Image J software, normalized to the mock group. Data are presented as mean values ± SD at least four biological replicates (n = 3 independent experiments). Statistical significance was determined by Log-rank test (A), Two-way ANOVA (B, C), One-way ANOVA (E) and unpaired Student’s t test (F). ns: not significant; * P < 0.05; ** P < 0.01; *** P < 0.001. (TIF) [file ppat.1014453.s006.tif]

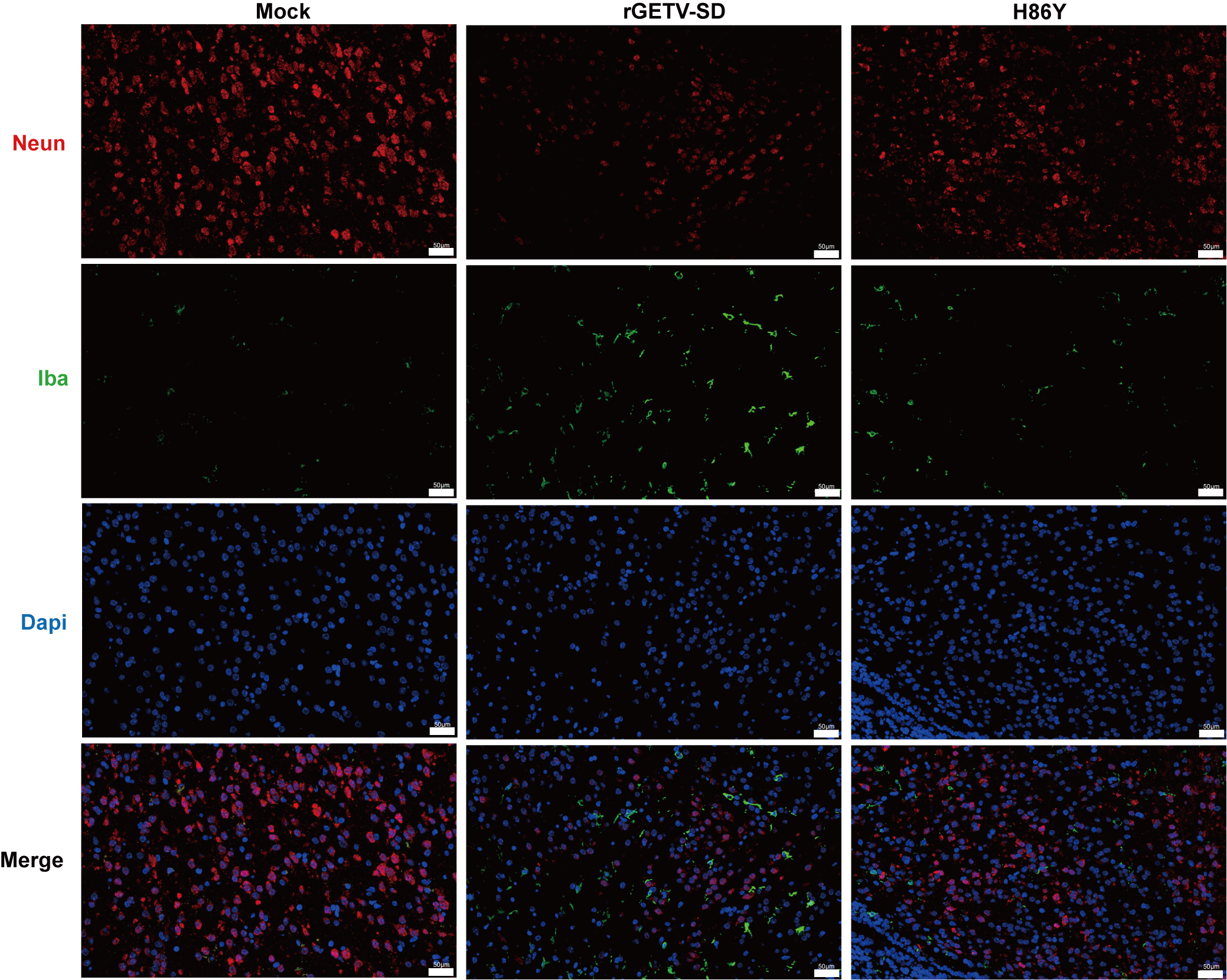

Supplement: S7 Fig — Neurons were labeled with anti-Neun antibody (red), microglia with anti-Iba1 antibody (green), and cell nuclei with DAPI (blue). Scale bar: 50 μm. Representative images were presented after similar results were obtained from t two independent experiments. (TIF) [file ppat.1014453.s007.tif]

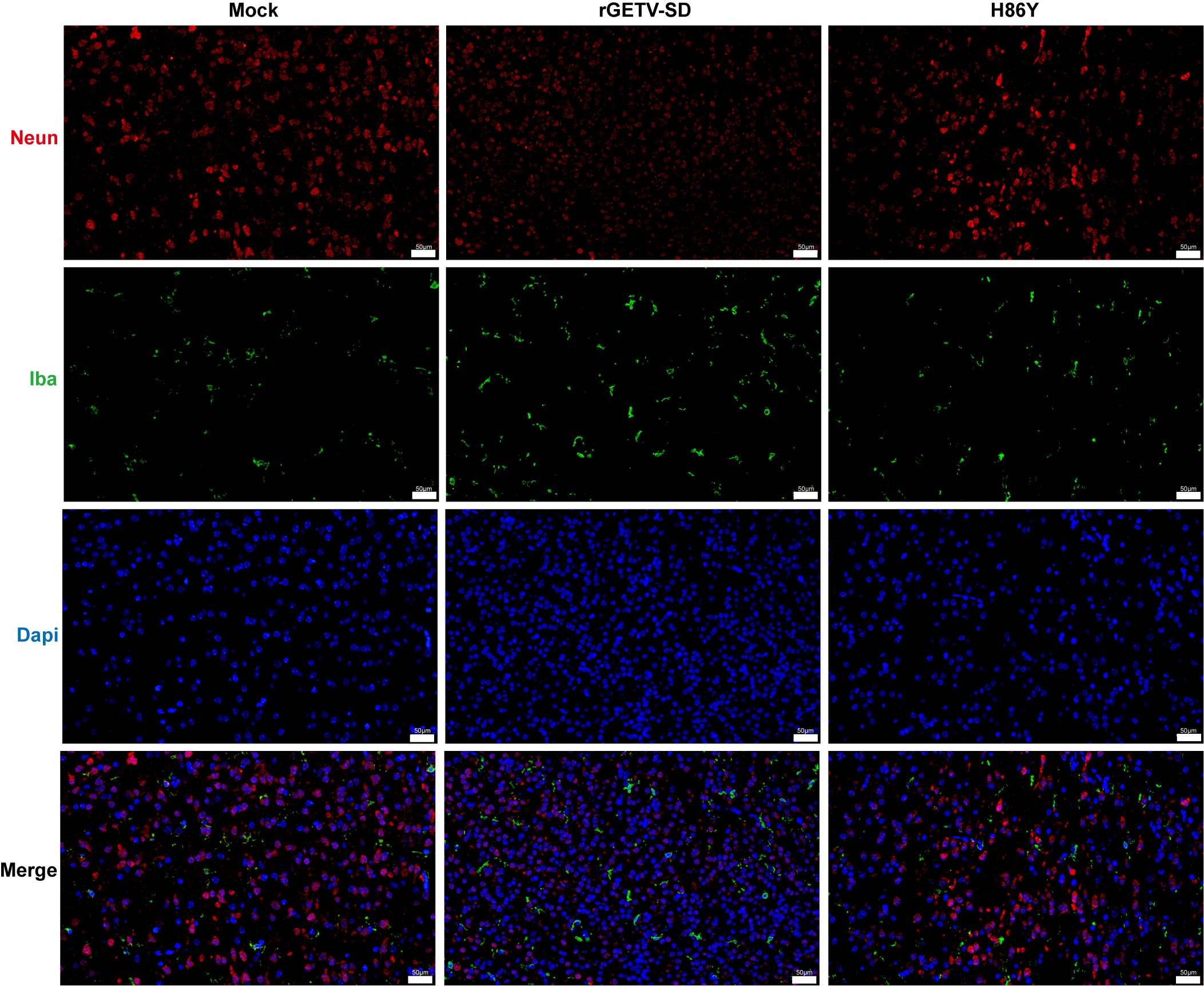

Supplement: S8 Fig — Neurons were labeled with anti-Neun antibody (red), microglia with anti-Iba1 antibody (green), and cell nuclei with DAPI (blue). Scale bar: 50 μm. Representative images were presented after similar results were obtained from two independent experiments. (TIF) [file ppat.1014453.s008.tif]

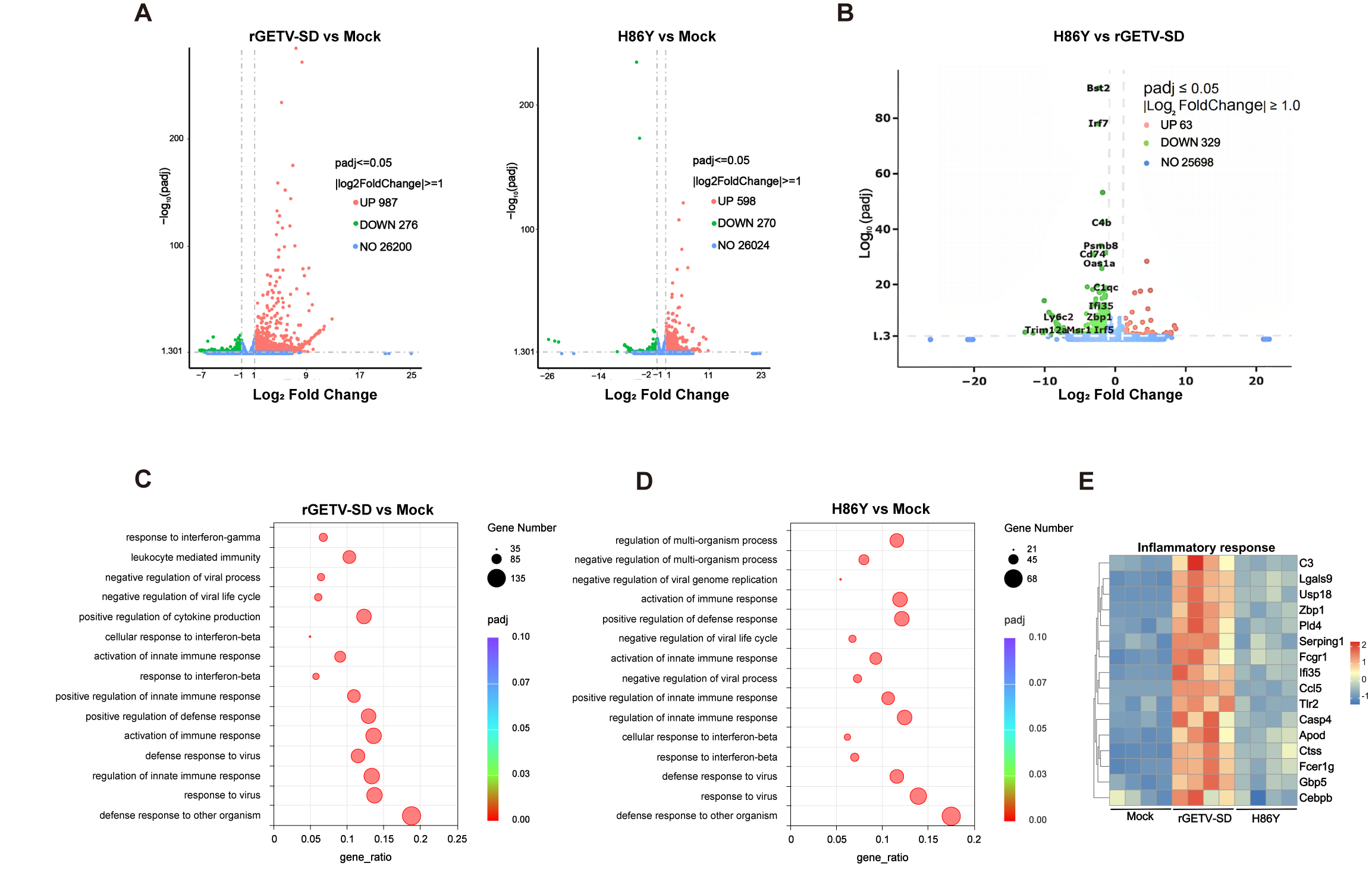

Supplement: S9 Fig — (A and B) Volcano plots indicating differentially regulated genes of rGETV-SD or H86Y infected mouse brains. (C and D) Top 15 Gene Ontology terms of up-regulated genes in rGETV-SD or H86Y versus mock-infected brains. (E) Heatmap analyses of expression of genes related to the inflammatory response. Heatmap shows Z-score normalized expression; red indicates above mean, blue below mean. (TIF) [file ppat.1014453.s009.tif]

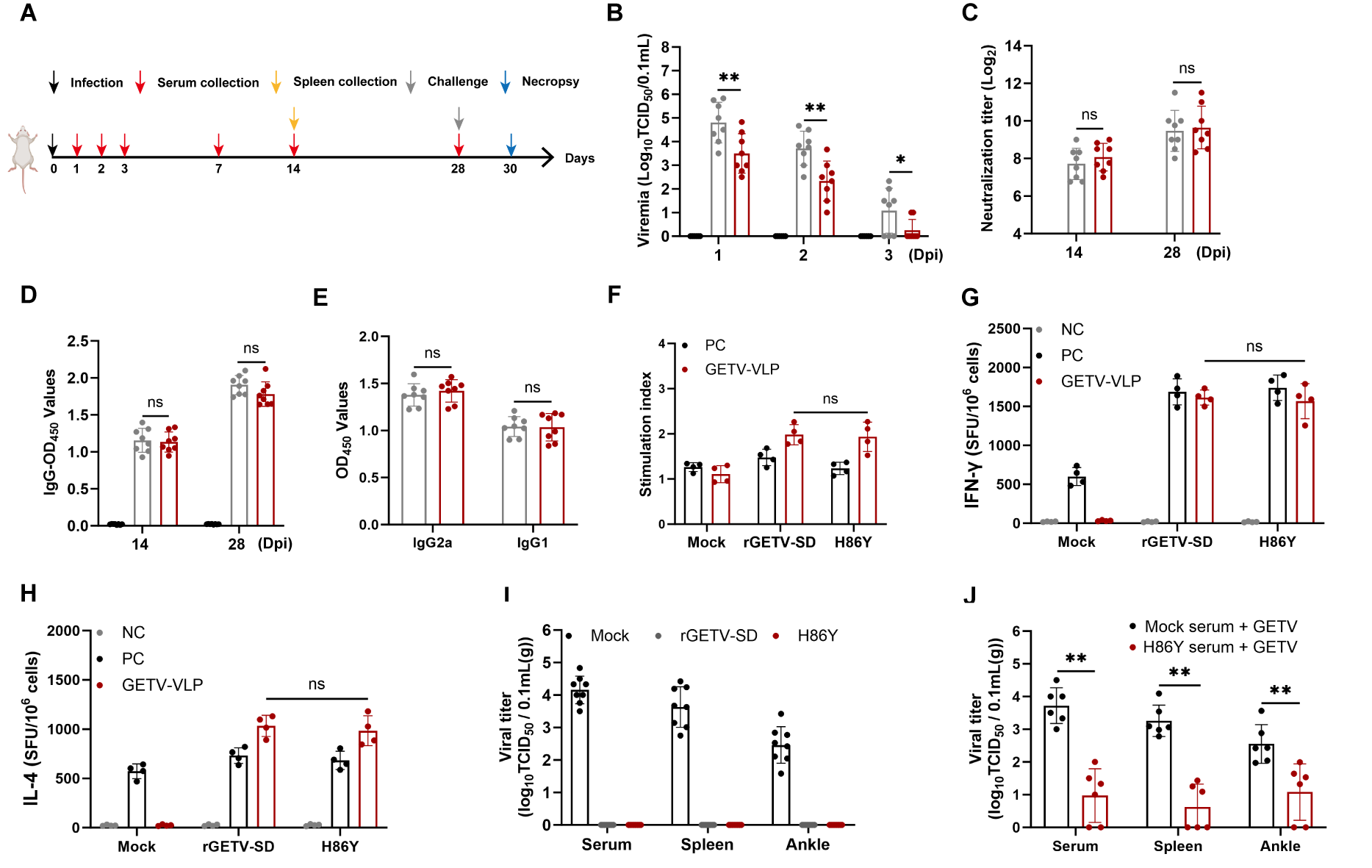

Supplement: S10 Fig — BALB/c adult mice were infected with 104 TCID50 of rGETV-SD or H86Y virus or DMEM via subcutaneous injection. At 28 dpi, the mice were subcutaneously challenged in the same site with 105 TCID50 of prevalent strain GETV SD2206 (n = 8). On day 2 post-challenge, the mice were euthanized. (A) Scheme of vaccination and challenge. (B) Mice viremia after infection. (C) Neutralizing antibody titers in sera at 14 dpi and 28 dpi determined using prevalent strain GETV SD2206. (D) GETV-specific IgG titers in serum at 14 and 28 dpi measured by ELISA performed using inactivated rGETV-SD or H86Y by formaldehyde and presented as OD450 values. (E) The OD450 values of IgG2a and IgG1 isotypes in sera of rGETV-SD or H86Y infected mice. (F-I) Two weeks after immunization, mouse spleens were collected, spleen cells were isolated and restimulated with recombinant GETV VLP protein (n = 4). (F) Specific splenocyte proliferation. RPMI 1640 medium was used as a negative control, and concanavalin A was used as a positive control. The stimulation index (SI) was calculated using the formula SI = (OD stimulant - OD 1640) / (OD control - OD 1640). (G and H) The spleen lymphocytes secreting IFN-γ and IL-4 were quantified by ELISPOT with both negative and positive controls. (I) Viremia and viral titers in spleen and ankle at 2 dpi. Means and SDs from at least four biological replicates are shown. (J) Passive transfer of immune serum protects mice from GETV challenge. Pooled sera collected from H86Y-immunized mice at 28 days post-immunization were injected intraperitoneally into naive recipient mice and control recipients received serum from mock-immunized mice once daily for two consecutive days. 24 hours later, all recipients were challenged subcutaneously with 105 TCID50 of GETV-SD2206. Viremia and viral titers in spleen and ankle at 2 dpi were determined by TCID50 (n = 2 independent experiments). Statistical significance was determined by Two-way ANOVA (B, C, D, F, G and H), unpaired Student’s t [file ppat.1014453.s010.tif]

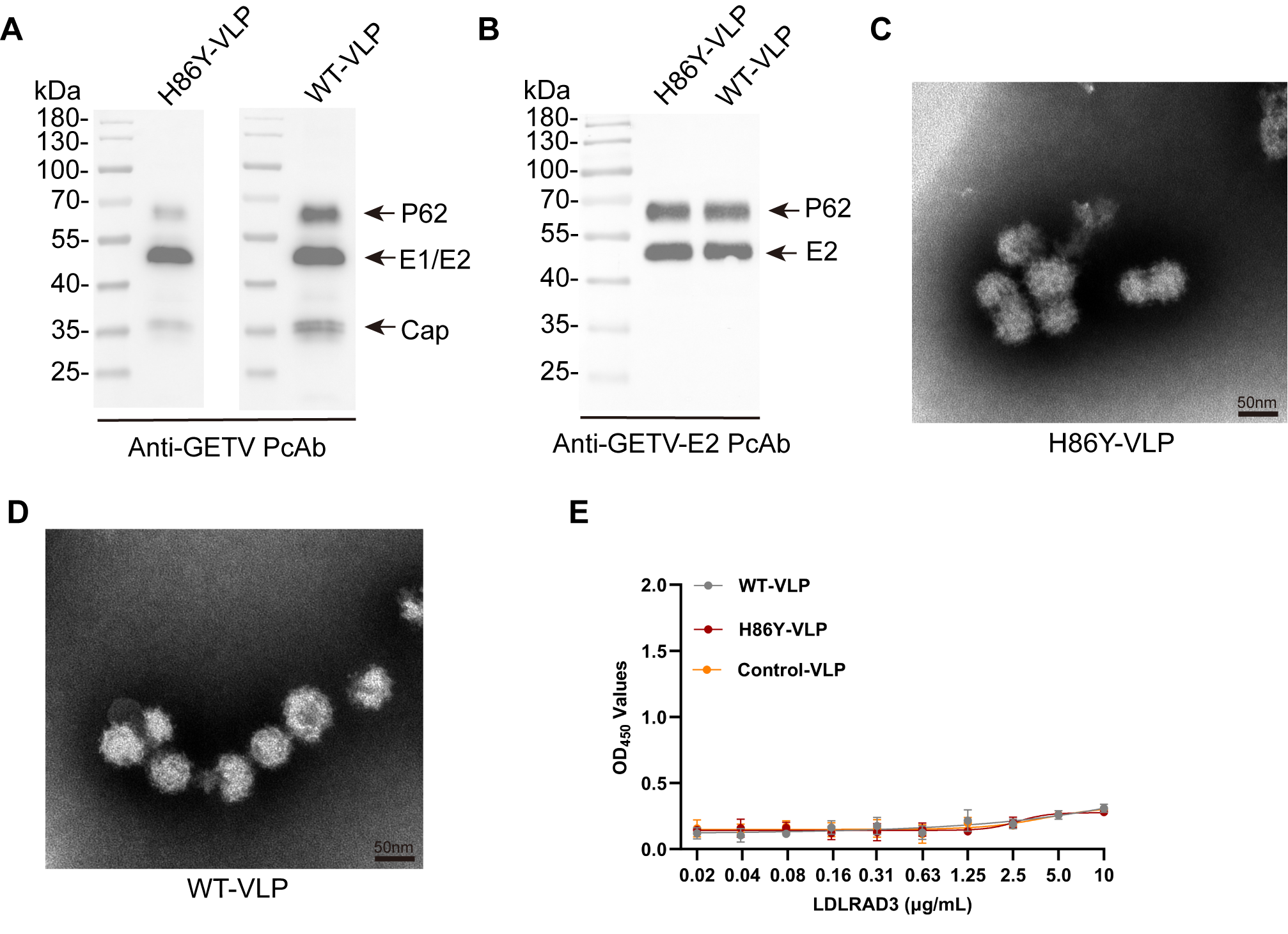

Supplement: S11 Fig — (A and B) Proteins present in purified VLPs of GETV were separated using western-blot with anti-GETV PcAb and anti-GETV-E2 PcAb polyclonal antibody. Molecular masses are indicated in kDa. PcAb, polyclonal antibody. (C and D) Electron micrographs of negatively stained purified VLPs of GETV. Scale bar is 50 nm. (E) ELISA-binding of WT-VLP, H86Y-VLP or control VLP to irrelevant receptor LDLRAD3. Representative images were presented after similar results were obtained from two independent experiments. (TIF) [file ppat.1014453.s011.tif]

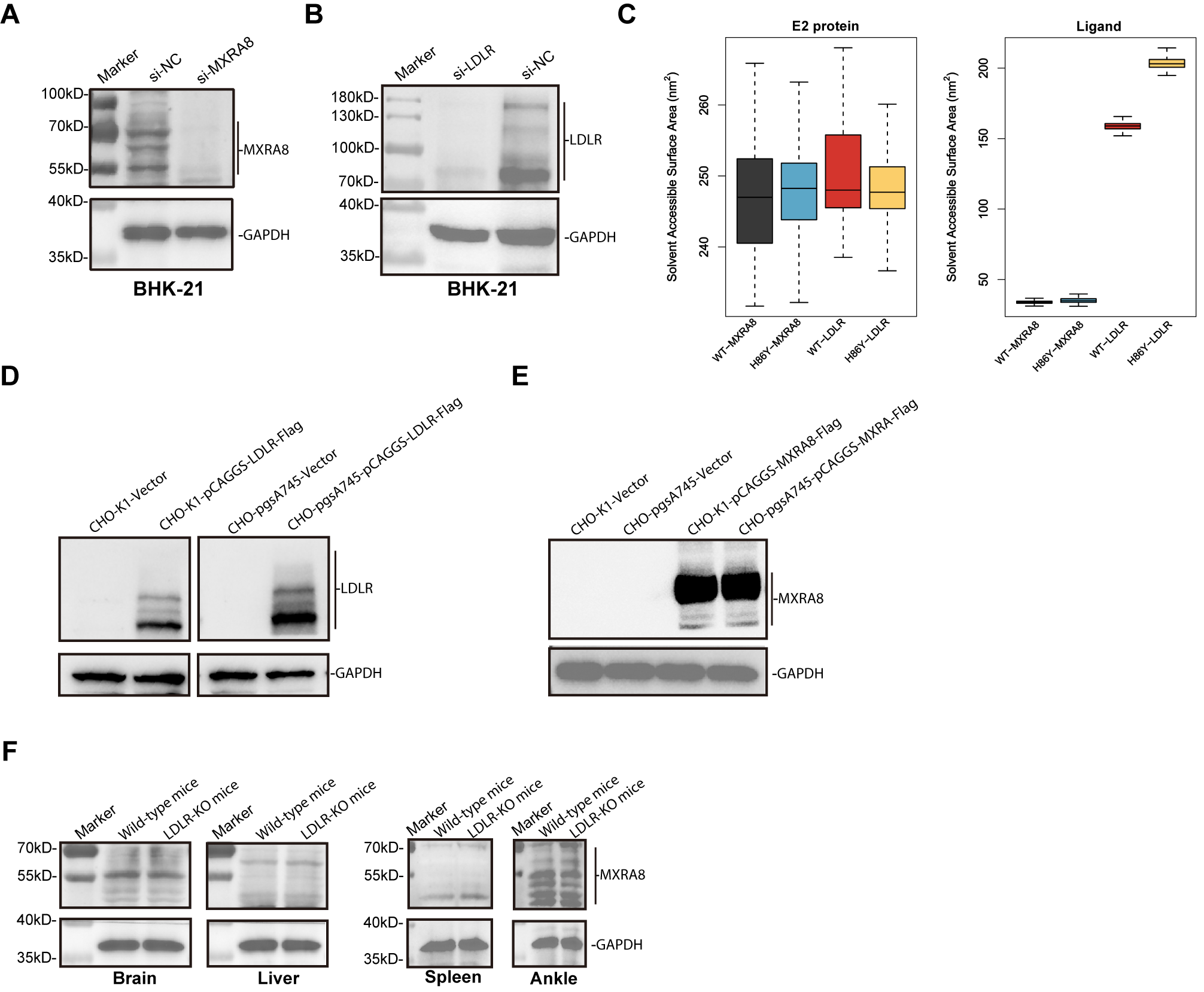

Supplement: S12 Fig — (A, B) BHK-21 cells were either transfected with siMXRA8 or siLDLR for 24 h and then identified by Western-blot. (C) Boxplots representing the distribution of ligand SASA over the simulation trajectories. (D-E) The endogenous expression level and over-expression level of LDLR or MXRA8 in CHO-K1 and CHO-pgsA745 cells. (F) The endogenous expression level of LDLR in tissues from both wild-type mice and LDLR-deficient mice. The experiment was performed twice with similar results, and representative micrographs are shown. (TIF) [file ppat.1014453.s012.tif]

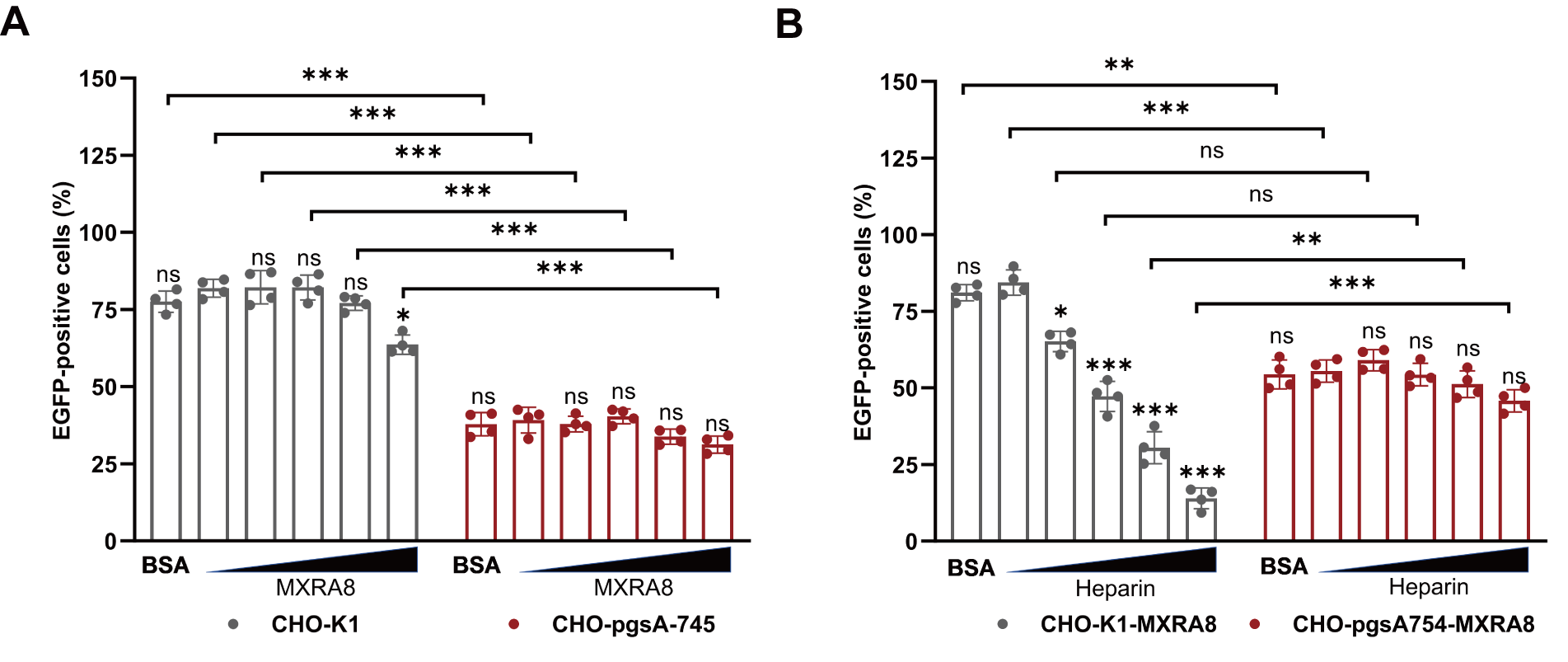

Supplement: S13 Fig — (A) Neutralization of WT-EGFP by MXRA8 (0, 0.5, 1, 10 µg/mL) in CHO-K1 and CHO-pgsA-745 cells by flow cytometry. (B) Neutralization of WT-EGFP by heparin (0, 10, 100, 500 and 1000 µg/mL) in CHO-K1 and CHO-pgsA-745 which both over-expressing MXRA8 receptor by flow cytometry. Means and SDs from at least four biological replicates (n = 3 independent experiments). Statistical significance was determined by Two-way ANOVA. ns: not significant; * P < 0.05; ** P < 0.01; *** P < 0.001. (TIF) [file ppat.1014453.s013.tif]

Fig 5D

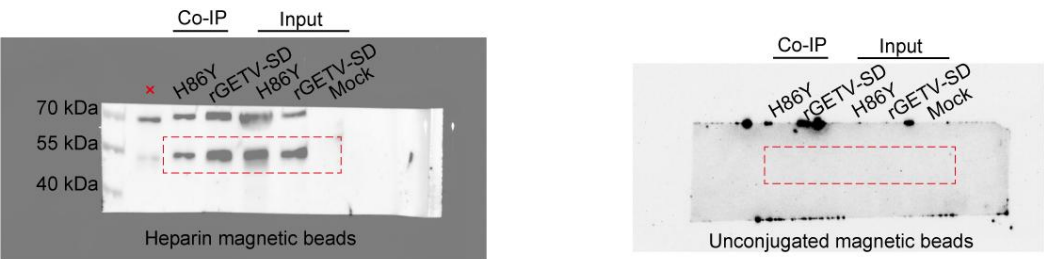

Fig 6M

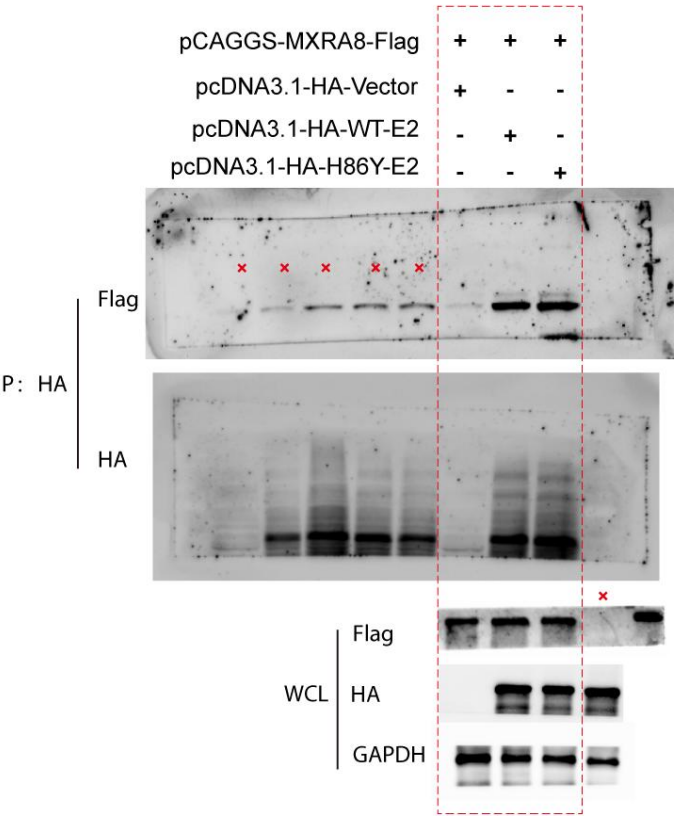

Fig 6N

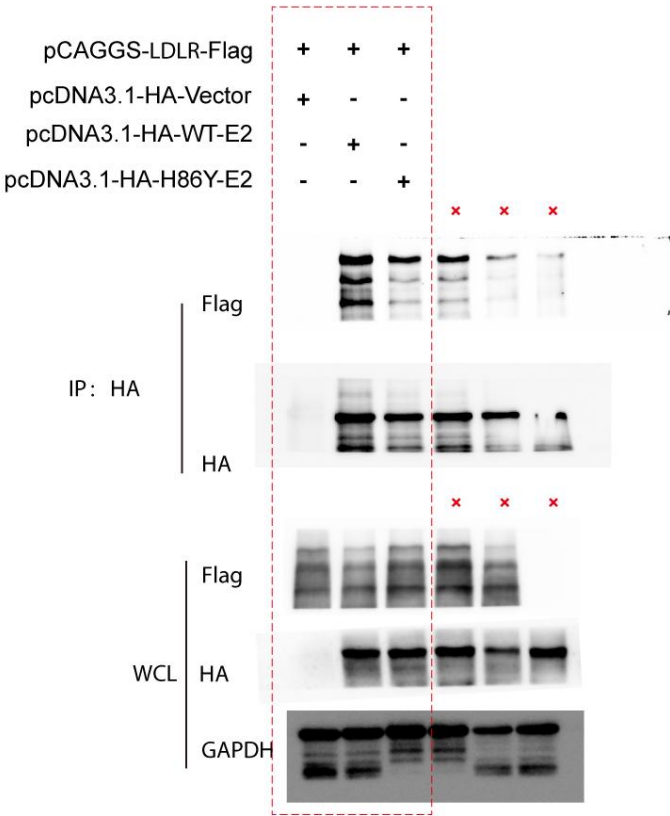

S11\_Fig

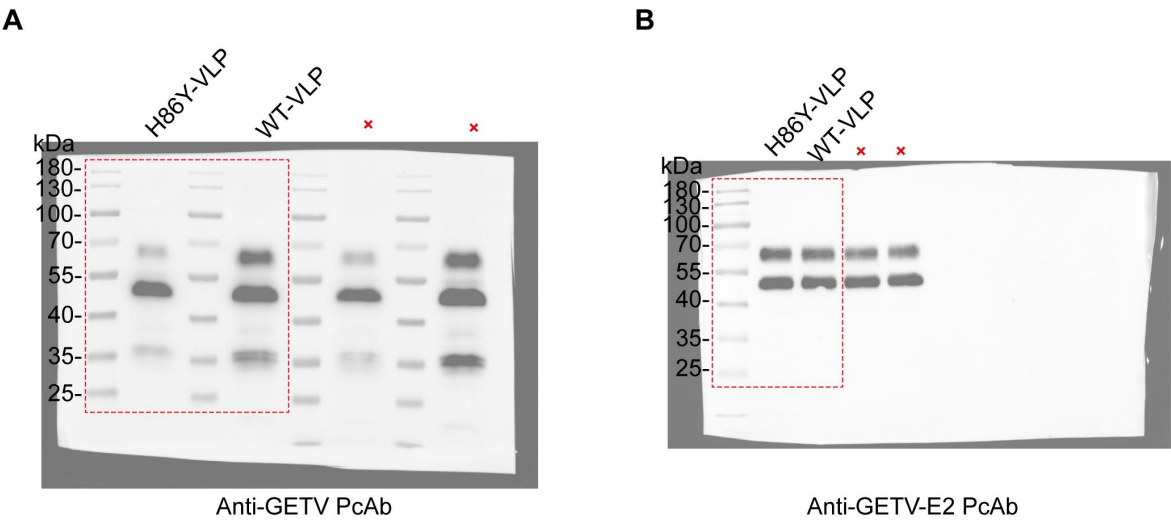

S12\_Fig

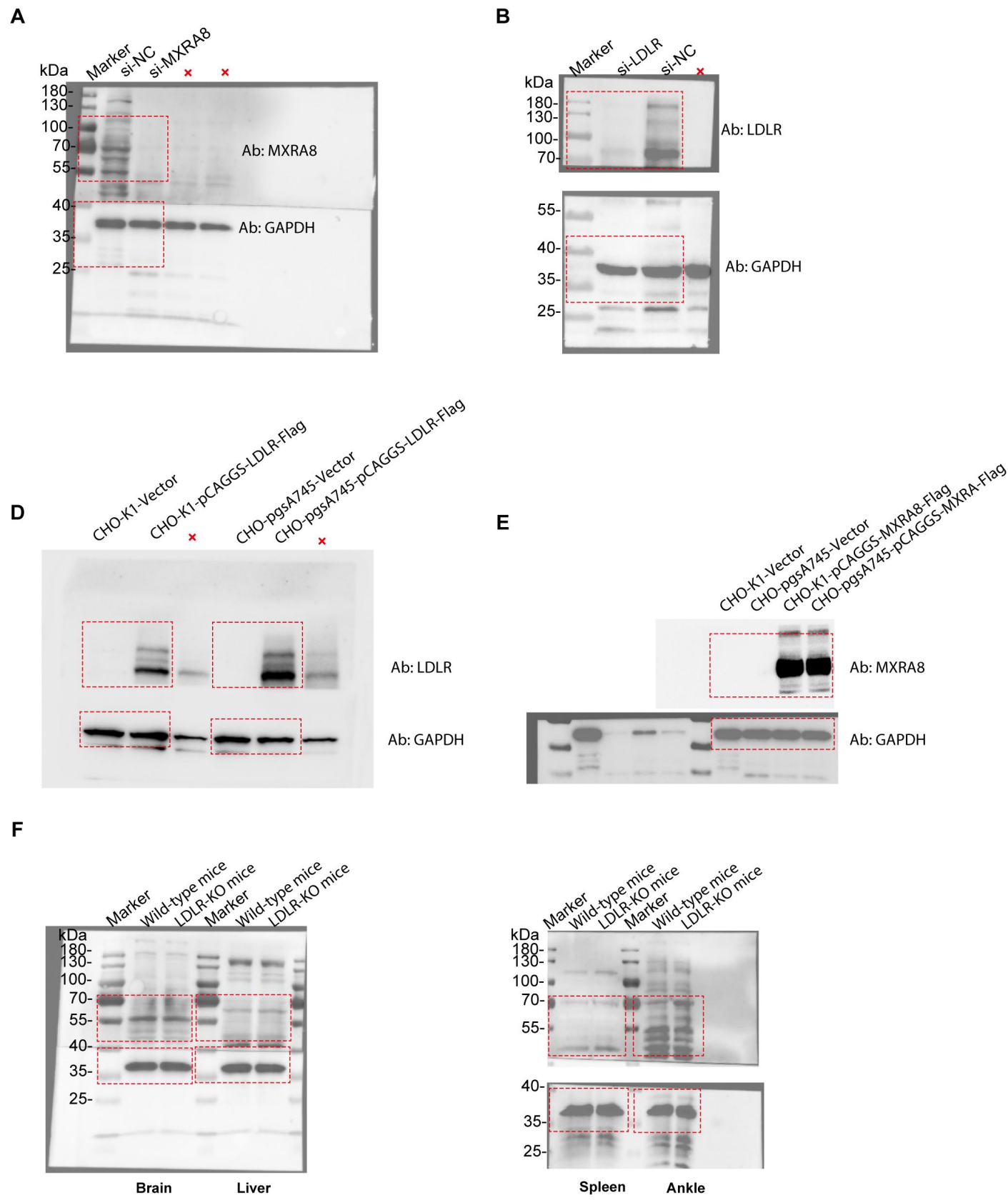

Supplement: S1 Raw Images — (PDF) [file ppat.1014453.s022.pdf]
