# Supplementary material for: The residue 86 of the Getah virus E2 glycoprotein mediates both glycosaminoglycan- and LDLR-dependent infection
Source: PLoS Pathog. 2026 Jul 31;22(7):e1014453. doi: 10.1371/journal.ppat.1014453 (PMC13426916; doi:10.1371/journal.ppat.1014453)
Supplement: S1 Table — (DOCX) [file ppat.1014453.s014.docx]

**S1 Table. Adaptive selection sites of GETV-E2 protein.**

| **Site** | **FUBAR**  **(Post.Pro)** | **MEME**  **(p-value)** | **FEL**  **(p-value)** | **SLAC**  **(p-value)** |
| --- | --- | --- | --- | --- |
| **H86Y** | 0.919 | 0.0747 | 0.0721 | - |
| **D323E** | 0.998 | 0.0124 | 0.0114 | - |
| **A368V** | 0.916 | - | 0.0897 | - |
