# Supplementary material for: The residue 86 of the Getah virus E2 glycoprotein mediates both glycosaminoglycan- and LDLR-dependent infection
Source: PLoS Pathog. 2026 Jul 31;22(7):e1014453. doi: 10.1371/journal.ppat.1014453 (PMC13426916; doi:10.1371/journal.ppat.1014453)
Supplement: S2 Table — (DOCX) [file ppat.1014453.s015.docx]

**S2 Table. Adaptive mutation sites of the GETV-E2 protein in different studies.**

| **Adaptive selection sites of GETV-E2 protein** | **Reference** |
| --- | --- |
| 86, 323, 368 | This study |
| 27, 86, 90, 207, 262, 269 | [2] |
| 86, 253, 323 | [21] |
| 4, 36, 41, 86, 248, 269, 323 | [3] |
