# Supplementary material for: The residue 86 of the Getah virus E2 glycoprotein mediates both glycosaminoglycan- and LDLR-dependent infection
Source: PLoS Pathog. 2026 Jul 31;22(7):e1014453. doi: 10.1371/journal.ppat.1014453 (PMC13426916; doi:10.1371/journal.ppat.1014453)
Supplement: S4 Table — (DOCX) [file ppat.1014453.s017.docx]

**S4 Table. Sequences of the primers for constructing infectious clone of GETV.**

| **Primer name** | **Primer sequence (5’−3’)** |
| --- | --- |
| CMV-F | CGTCTAGAGACATTGATTATTGACTAGTTATTAATAGTAATCA |
| CMV-R | CGTTAATTAAAGCTCTGCTTATATAGACCTCCCA |
| S1-F | CGTTAATTAAATGGCGGACGTGTGACATCA |
| S1-R | CGTCGCGAGTCGTCACCATGTT |
| S2-F | CATCGCGATCTGGTGGCCAGTG |
| S2-R | CACAGATCTGCTCGTTAGCATCTTGGA |
| S3-F | CGAGATCTGCCTGTACGCCCTA |
| S3-R | CTCACCGAATTTAAATCTTGTACCG |
| S4-1-F | TACGGTACAAGATTTAAATTCGGTG |
| S4-1-R | GTCATCGTGGCTTCGAGCAGGTCGTA |
| S4-2-F | TACGACCTGCTCGAAGCCACGATGAC |
| S4-2-R | TAGGGCCCACAAGCGCACGGGT |
| S5-F | CTACCCGTGCGCTTGTGGGCCC |
| S5-R | CGATCGATTTTTTTTTTTTTTTTTTTTTGTAAAATATTAAAAAAAC |
| PCR1-SGP-F | TACGGTACAAGATTTAAATTCGGTG |
| PCR1-SGP-R | AGTGTCTGTAATACGTGGTCTTTAG |
| PCR2-SGP-F | TCTAAAGACCACGTATTACAGACACTCTCGAGATCGAGGGCGCGCCATGAATTACATT |
| PCR2-SGP-R | CTACGCGTCCTTTGTTGTCGAA |
| WT/H86Y-PCR1-F | AGGCTTCCGATGGCATGATAAAAATCCAGGTCGCAGCGCAAATT |
| WT-PCR1-R | GCGCACGTACCGGAAGTATGCACTTGTAAAGAATCCCGG |
| H86Y-PCR1-R | GCGCACGTACCGGAAGTATaCACTTGTAAAGAATCCCGG |
| WT-PCR2-F | CCGGGATTCTTTACAAGTGCATACTTCCGGTACGTGCGC |
| H86Y-PCR2-F | CCGGGATTCTTTACAAGTGtATACTTCCGGTACGTGCGC |
| WT/H86Y-PCR2-R | CCTTCAGTTGTCAGCTGGGCCCACAAGCGCACGGGTGGGTTGTTTCCC |
| EGFP-F | AGCTCGAGATGGTGAGCAAGGGCGAGGA |
| EGFP-R | AGGGCGCGCCTTACTTGTACAGCTCGTCCA |
