# Supplementary material for: The residue 86 of the Getah virus E2 glycoprotein mediates both glycosaminoglycan- and LDLR-dependent infection
Source: PLoS Pathog. 2026 Jul 31;22(7):e1014453. doi: 10.1371/journal.ppat.1014453 (PMC13426916; doi:10.1371/journal.ppat.1014453)
Supplement: S5 Table — (DOCX) [file ppat.1014453.s018.docx]

**S5 Table. Sequences of the primers used for PCR.**

| **Primer name** | **Primer sequence (5’−3’)** |
| --- | --- |
| GETV-E1-F | CAGGACAACCAGGGAGGTTC |
| GETV-E1-R | CGTGAACAGTACCGGACGAA |
| GAPDH-BHK-21-F | GGTGGGAATGGGTCAGAAG |
| GAPDH-BHK-21-R | AGCTCATTGTAGAAGGTG TGG |
| GAPDH-ST-F | CCTTCCGTGTCCCTACTGCCAAC |
| GAPDH-ST-R | GACGCCTGCTTCACCACCTTCT |
| GAPDH-Vero-F | TCCTTGGAGGCCATGTGGGCCAT |
| GAPDH-Vero-R | TGATGACATCAAGAAGGTGGTGAAG |
| β-actin-C6/36-F | GGAGAAGATCTGGCATCACA |
| β-actin-C6/36-R | TGTCATCTTCTCGCGGTTAG |
| pCA-mes-MXRA8-F | gtctcatcattttggcaaagaattcgccaccATGGAACTGCTGTCCTGTGTCTTG |
| pCA-mes-MXRA8-R | atccttgtaatcacctccTTTGCAGTACTCCTTCCTGAACTCTTTATCC |
| pCA-mes-LDLR-F | gtctcatcattttggcaaagaattcgccaccATGCGCACCGCGGATCT |
| pCA-mes-LDLR-R | atccttgtaatcacctccTGCCACATCATCCTCCAGGCT |
| si-mes-MXRA8 | GUGGAAACUGGUGCUUCUU |
| si-mes-LDLR | GAGCAUCAACAGCAUAAAC |
| Mouse-TNF-α-F | AGAAACACAAGATGCTGGGACAGT |
| Mouse-TNF-α-R | CCTTTGCAGAACTCAGGAATGG |
| Mouse-Ccl5-F | CTCCCTGCTGCTTTGCCTAC |
| Mouse-Ccl5-R | CGGTTCCTTCGAGTGACAAACA |
| Mouse-IL-6-F | TCCATCCAGTTGCCTTCTTG |
| Mouse-IL-6-R | GGTCTGTTGGGAGTGGTATC |
| Mouse-β-actin-F | GAGACCTTCAACACCCCAGC |
| Mouse-β-actin-R | ATGTCACGCACGATTTCCC |
