# Supplementary material for: The residue 86 of the Getah virus E2 glycoprotein mediates both glycosaminoglycan- and LDLR-dependent infection
Source: PLoS Pathog. 2026 Jul 31;22(7):e1014453. doi: 10.1371/journal.ppat.1014453 (PMC13426916; doi:10.1371/journal.ppat.1014453)
Supplement: S6 Table — (DOCX) [file ppat.1014453.s019.docx]

**S6 Table. The sequencing results of H86Y and rGETV-SD.**

| **Virus strian** | **Position** | **Sequencing type** | **nucleotide** | **Evidence** | **Frequency** |
| --- | --- | --- | --- | --- | --- |
| H86Y-BHK-P15 | 8778 | SNP | T | T:27541 C:81 | 0.29% |
| H86Y-Mouse | 8778 | SNP | T | T:3639 C:4 | 0.11% |
| H86Y-BHK-P1 | 8778 | Sanger | T |  |  |
| H86Y-BHK-P7 | 8778 | Sanger | T |  |  |
| H86Y-BHK-P15 | 8778 | Sanger | T |  |  |
| rGETV-SD-BHK-P1 | 8778 | Sanger | C |  |  |
| rGETV-SD-BHK-P7 | 8778 | Sanger | C |  |  |
| rGETV-SD-BHK-P15 | 8778 | Sanger | C |  |  |

SNP: single nucleotide polymorphism. A mutation is considered reliable only if its frequency exceeds 5%.
