# Supplementary material for: The residue 86 of the Getah virus E2 glycoprotein mediates both glycosaminoglycan- and LDLR-dependent infection
Source: PLoS Pathog. 2026 Jul 31;22(7):e1014453. doi: 10.1371/journal.ppat.1014453 (PMC13426916; doi:10.1371/journal.ppat.1014453)
Supplement: S7 Table — (DOCX) [file ppat.1014453.s020.docx]

**S7 Table. Statistics of depth and coverage information of H86Y.**

| **Sequence name** | **Average depth** | **Median** | **Coverage%** | **Cov 10x %** | **Cov 100x %** |
| --- | --- | --- | --- | --- | --- |
| H86Y-BHK-P15 | 25059.10 | 24608.0 | 100.00 | 100.00 | 99.96 |
| H86Y-Mouse | 3305.36 | 3080.0 | 100.00 | 100.00 | 99.64 |

Avg depth: Average mapping depth

Median: Median mapping depth

Coverage%: Mapping coverage (%)

Cov 10X: Percentage of reference sequence covered at ≥10× depth

Cov 100X: Percentage of reference sequence covered at ≥100× depth
